# Neutralization of SARS-CoV-2 Omicron by BNT162b2 mRNA vaccine–elicited human sera

**DOI:** 10.1126/science.abn7591

**Published:** 2022-01-18

**Authors:** Alexander Muik, Bonny Gaby Lui, Ann-Kathrin Wallisch, Maren Bacher, Julia Mühl, Jonas Reinholz, Orkun Ozhelvaci, Nina Beckmann, Ramón de la Caridad Güimil Garcia, Asaf Poran, Svetlana Shpyro, Andrew Finlayson, Hui Cai, Qi Yang, Kena A. Swanson, Özlem Türeci, Uğur Şahin

**Affiliations:** 1 BioNTech, An der Goldgrube 12, 55131 Mainz, Germany.; 2 BioNTech US, 40 Erie Street, Cambridge, MA 02139, USA.; 3 Pfizer, 401 N Middletown Rd., Pearl River, NY 10960, USA.; 4 HI-TRON – Helmholtz Institute for Translational Oncology Mainz by DKFZ, Obere Zahlbacherstr. 63, 55131 Mainz, Germany.; 5 TRON gGmbH – Translational Oncology at the University Medical Center of the Johannes Gutenberg University, Freiligrathstraße 12, 55131 Mainz, Germany.

## Abstract

The globally circulating severe acute respiratory syndrome coronavirus 2 (SARS-CoV-2) variant of concern Omicron (B.1.1.529) has a large number of mutations, especially in the spike protein, indicating that recognition by neutralizing antibodies may be compromised. We tested Wuhan (Wuhan-Hu-1 reference strain), Beta (B.1.351), Delta (B.1.617.2), or Omicron pseudoviruses with sera of 51 participants who received two or three doses of the messenger RNA (mRNA)–based COVID-19 vaccine BNT162b2. After two doses, Omicron-neutralizing titers were reduced >22-fold compared with Wuhan-neutralizing titers. One month after the third vaccine dose, Omicron-neutralizing titers were increased 23-fold relative to their levels after two doses and were similar to levels of Wuhan-neutralizing titers after two doses. The requirement of a third vaccine dose to effectively neutralize Omicron was confirmed with sera from a subset of participants using live SARS-CoV-2. These data suggest that three doses of the mRNA vaccine BNT162b2 may protect against Omicron-mediated COVID-19.

## Vaccination protects against Omicron

The Omicron (B.1.1.529) variant of concern has proven more transmissible and infectious than the original severe acute respiratory syndrome coronavirus 2 (SARS-CoV-2) virus. Muik *et al*. investigated whether the BioNTech/Pfizer BNT162b2 messenger RNA (mRNA) vaccine is effective against the Omicron variant. Sera were collected from two groups of clinical trial subjects: those who received the primary two-dose BNT162b2 vaccine series (spaced 21 days apart) and those who received a third, “booster” vaccine 6 to 18 months after the second BNT162b2 dose. Individuals who received only two doses had a low ability to neutralize the Omicron variant, whereas a third shot of BNT162b2 significantly improved antibody recognition of Omicron. These data suggest that three BNT162b2 mRNA doses are likely required to protect against Omicron-driven COVID-19. —PNK

## Teaser

Sera from individuals vaccinated with three doses of the Pfizer/BioNtech mRNA vaccine BNT162b2 can neutralize the SARS-CoV-2 Omicron variant.

Since the first reports of severe acute respiratory syndrome coronavirus 2 (SARS-CoV-2) in humans in December 2019, numerous genetically distinct lineages have evolved. Among those, variants of concern (VOCs), especially the Alpha (B.1.1.7) and Delta (B.1.617.2) variants, were associated with increased viral transmissibility and sparked new waves of infection, with Delta [first designated a VOC on 11 May 2021 (*1*)] quickly becoming a globally dominant variant (*2*). On 26 November 2021, a new VOC—Omicron (B.1.1.529)—was reported by the World Health Organization (WHO) (*3*). Omicron is a highly divergent variant and harbors a previously unseen number of mutations in its spike (S) glycoprotein (*4*). Fifteen mutations are located in the receptor binding domain and another eight mutated sites are found in the N-terminal domain, both being immunodominant targets of neutralizing antibodies elicited by COVID-19 vaccines or by SARS-CoV-2 infection (*5*, *6*). Some amino acid changes [Δ69/70, T95I, G142D, Δ145, K417N, T478K, N501Y, and P681H (T, Thr; I, Ile; G, Gly; D, Asp; K, Lys; N, Asn; Y, Tyr; P, Pro; H, His)] are shared mutations also found in the Alpha, Beta (B.1.351), Gamma (P.1), or Delta VOCs and were described to lead to increased transmissibility, as well as to a typically mild partial escape from vaccine-induced humoral immunity (*7*–*10*).

The BNT162b2 COVID-19 mRNA vaccine contains lipid nanoparticle–formulated mRNA that encodes the SARS-CoV-2 S glycoprotein from the parental Wuhan reference strain (Wuhan-Hu-1) (*11*). Administration of two 30-μg doses of BNT162b2 was shown to have 95% efficacy in a phase 3 trial (*12*) and to elicit strong antibody responses, effectively neutralizing the parental strain as well as diverse SARS-CoV-2 VOCs (*13*–*15*). Because neutralizing antibody titers are strongly predictive of the degree of immune protection against symptomatic SARS-CoV-2 infection (*16*), it is important to understand the effect of the new mutations in Omicron on recognition by neutralizing antibodies in convalescent and vaccinated individuals.

To evaluate whether BNT162b2-elicited antibodies (*11*) are capable of neutralizing the Omicron variant, we used two orthogonal test systems: a pseudovirus-neutralization test (pVNT) that has been shown to be in close concordance with live SARS-CoV-2–neutralization assays (*17*, *18*), as well as a live SARS-CoV-2–neutralization test (VNT). For the former, we generated vesicular stomatitis virus (VSV)–SARS-CoV-2-S pseudoviruses bearing the S proteins of the Wuhan strain, Omicron, Beta [as a benchmark for partially reduced neutralization (*7*) without major impact on effectiveness (*19*, *20*)], or Delta (the predominant strain until mid-December 2021). BNT162b2 immune sera from vaccinated individuals between 20 and 72 years of age (with more than one-third being ≥56 years of age; table S1) were obtained from different clinical trials—the phase 1/2 trial BNT162-01 (NCT04380701), the phase 2 rollover trial BNT162-14 (NCT04949490) conducted in Germany, and the global phase 2 trial BNT162-17 (NCT05004181) (see materials and methods). Neutralizing titers against VSV-SARS-CoV-2-S pseudoviruses were analyzed with serum drawn from 32 participants from the BNT162-01 trial 21 days (median of 22 days; range: 19 to 23 days) after two doses of BNT162b2 (median time from dose one to dose two: 21 days; range: 19 to 23 days; table S1) and with serum drawn from 30 participants from the BNT162-14 (*n* = 11) and BNT162-17 (*n* = 19) trials 1 month (median: 28 days; range: 26 to 30 days) after the third dose of BNT162b2 (median time from dose two to dose three: 219 days; range: 180 to 342 days). Eleven of the individuals in this analysis were rolled over from the BNT162-01 into the BNT162-14 trial and were included in a longitudinal analysis of neutralizing antibody responses against Wuhan or Omicron variant pseudovirus. These individuals were immunized with a third dose of BNT162b2, with sera collected (i) 21 days after the second dose (median: 21 days; range: 19 to 23 days), (ii) directly before the third dose (median: 256 days after dose two; range: 180 to 342 days), and (iii) 1 month (28 days) after the third dose.

**Fig. 1.**
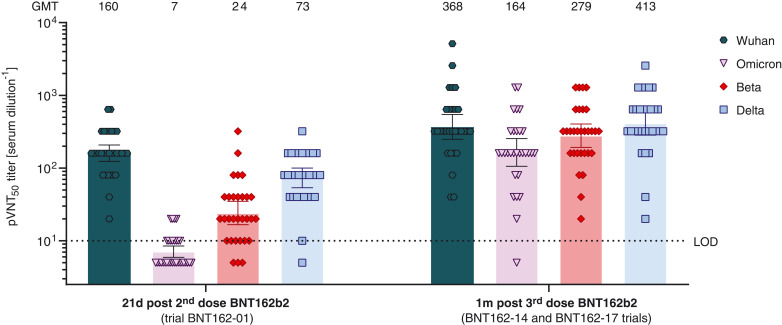
50% pseudovirus-neutralization titers (pVNT_50_) of sera from vaccine recipients, collected after two or three doses of BNT162b2 against VSV-SARS-CoV-2-S pseudovirus bearing the Wuhan, Omicron, Beta, or Delta variant S protein. *N* = 32 serum specimens from participants in trial BNT162-01, drawn 21 days after dose two, and *n* = 30 serum specimens from participants in the BNT162-14 (*n* = 11) and BNT162-17 (*n* = 19) trials, drawn 1 month after dose three, were tested. Each specimen was tested in duplicate, and geometric mean 50% pseudovirus-neutralizing titers (GMTs) were plotted. Below the limit of detection (LOD), LOD/2 values were plotted. Group GMTs (values) and 95% confidence intervals are indicated.

After two doses of BNT162b2, geometric mean neutralization titers (GMTs) against Omicron pseudovirus were reduced 22.8-fold compared with those for the Wuhan reference pseudovirus (Fig. 1; GMT of 7 versus 160). Twenty of 32 immune serum specimens displayed no detectable neutralizing activity against Omicron (table S2). By contrast, the majority of sera neutralized Beta and Delta pseudoviruses with GMTs of 24 and 73, respectively. This corresponds to 6.7- and 2.2-fold reductions in neutralization activity compared with the Wuhan pseudovirus and is in line with previous reports (*11*, *14*, *15*, *21*).

One month after the third BNT162b2 dose, neutralizing GMTs against the Omicron variant pseudovirus increased 23.4-fold relative to their levels 21 days after the second dose (GMT of 164 versus 7) and were comparable to neutralizing GMTs against the reference Wuhan pseudovirus at 21 days after two doses of BNT162b2 (GMT of 164 versus 160). Twenty-nine of 30 serum specimens were capable of neutralizing the Omicron pseudovirus (table S3). The third dose of BNT162b2 also increased neutralizing activity against Beta, Delta, and Wuhan pseudoviruses, with GMTs of 279, 413, and 368, respectively.

For 11 individuals included in the aforementioned analyses, a longitudinal analysis of neutralizing titers against Omicron and Wuhan pseudovirus was performed. Twenty-one days after dose two, sera exhibited a 21.4-fold reduction in GMT against the Omicron variant compared with the Wuhan reference pseudovirus (fig. S1; GMT of 7 versus 150). Before receiving the third dose of BNT162b2 (at a median 256 days after dose two), neutralizing titers against the Wuhan pseudovirus were considerably reduced (GMT of 13), whereas the Omicron-specific titers were below the limit of detection. Consistent with the larger serum panel, the third dose of BNT162b2 resulted in a significant increase in neutralizing titers against the Wuhan pseudovirus (GMT of 320) and a 25.8-fold increase in neutralizing titers against Omicron 1 month after dose three compared with 21 days after dose two (GMT of 181 versus 7).

**Fig. 2.**
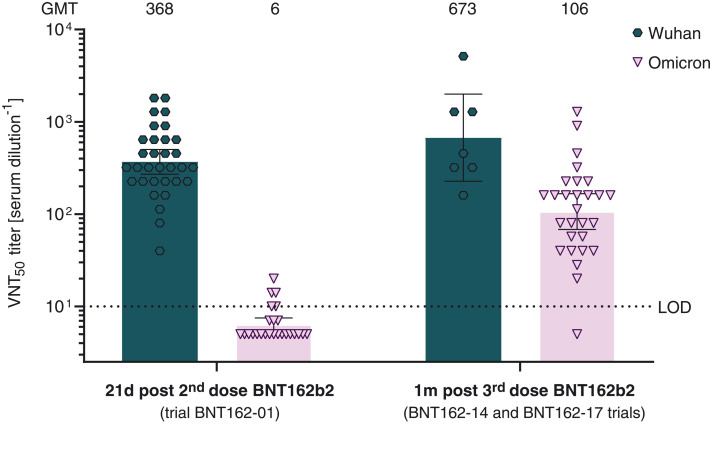
50% live SARS-CoV-2–neutralization titers (VNT_50_) of sera from vaccine recipients, collected after two or three doses of BNT162b2. Sera from participants in trial BNT162-01, drawn 21 days after dose two, were tested for neutralization against SARS-CoV-2 Wuhan (*n* = 32) and Omicron (*n* = 25), respectively. Sera from participants in the BNT162-14 and BNT162-17 trials, drawn 1 month after dose three, were tested for neutralization against SARS-CoV-2 Wuhan (*n* = 7 from BNT162-14) and Omicron (total *n* = 28: 11 from BNT162-14 and 17 from BNT162-17), respectively. Each specimen was tested in duplicate, and geometric mean 50% SARS-CoV-2–neutralizing titers (GMTs) were plotted. Below the limit of detection (LOD), LOD/2 values were plotted. Group GMTs (values) and 95% confidence intervals are indicated.

Sera from a subset of trial participants were analyzed with the second neutralization assay using live SARS-CoV-2 Wuhan and Omicron virus. Serum samples from 32 and 25 participants in trial BNT162-01, drawn 21 days after dose two, and from 7 and 28 participants in the BNT162-14 (*n* = 7 and 11) and BNT162-17 (*n* = 0 and 17) trials, drawn 1 month after dose three, were tested for neutralization against SARS-CoV-2 Wuhan and Omicron, respectively. Neutralizing GMTs against live SARS-CoV-2 Omicron were reduced 61.3-fold relative to those against the Wuhan reference strain (Fig. 2; GMT of 6 versus 368) at 21 days after two doses of BNT162b2. Seventeen of 25 serum specimens displayed no detectable neutralizing activity against Omicron (table S4). One month after the third BNT162b2 dose, neutralizing GMTs against Omicron were increased 17.6-fold compared with neutralizing GMTs at 21 days after the second dose (GMT of 106 versus 6) and were reduced 3.4-fold compared with those against the Wuhan reference at 21 days after two doses of BNT162b2 (GMT of 106 versus 368). Twenty-seven of 28 post–dose three serum specimens neutralized live SARS-CoV-2 Omicron (table S5).

The observed SARS-CoV-2–neutralizing GMTs correlated positively with the neutralizing GMTs against VSV-SARS-CoV-2-S pseudoviruses (fig. S2).

BNT162b2 vaccination induces strong polyepitopic T cell responses, directed against multiple epitopes spanning the length of the S protein (*11*). To assess the risk of immune evasion of CD8^+^ T cell responses by Omicron, we investigated a set of human leukocyte antigen class I–restricted T cell epitopes from the Wuhan S protein sequence that were reported as immunogenic in the Immune Epitope Database (IEDB) (*n* = 244; see materials and methods). Despite the multitude of mutations in the Omicron S protein, 85.3% (*n* = 208) of the described class I epitopes were not affected on the amino acid sequence level, indicating that the targets of most T cell responses elicited by BNT162b2 may still be conserved in the Omicron variant (fig. S3).

In summary, our data indicate that two doses of the BNT162b2 mRNA vaccine may not be sufficient to protect against infection with the Omicron variant. In both neutralization assay platforms, we observed a substantial reduction in neutralizing activity for immune sera drawn 21 days after the primary two-dose series of BNT162b2, confirming preliminary reports that describe a 20- to 40-fold reduction in titers (*22*, *23*). Both assays also showed that a third dose of BNT162b2 boosts Omicron-neutralization capability to robust levels. In the pseudovirus assay, Omicron-neutralization titers after three doses reach a level similar to that observed for Wuhan-neutralizing titers after two doses, whereas in the live SARS-CoV-2 assay, Omicron-neutralizing GMTs after dose three were reduced 3.4-fold relative to Wuhan-neutralizing GMTs after two doses. The observed variability in specific titers and fold differences between nonreplicating pseudovirus– and replicating live virus–neutralization assay platforms as well as different SARS-CoV-2 strains are not unexpected. Notably, the overall trends are similar and demonstrate that a third dose of BNT162b2 augments antibody-based immunity against Omicron, in line with previous observations that a third vaccination broadens humoral immune responses against VOCs (*24*).

In the analysis presented here, we have evaluated and compared serum panels from different clinical trials with a limited sample size. For BNT162-01 trial participants, the first two doses of BNT162b2 were separated by 21 days (median: 21 days; range: 19 to 23 days), but the time elapsed between the second and third doses was not consistent across participants. Recent reports indicate that a longer interval (>42 days) between the first and second doses improves immunogenicity, potentially resulting in a more favorable outcome (*25*). Future analyses are needed to evaluate antibody persistence.

Neutralizing antibodies represent a first layer of adaptive immunity against COVID-19. T cell responses play a vital role as a second layer of defense, in particular in the prevention of severe COVID-19 (*26*). CD8^+^ T cell responses in individuals vaccinated with BNT162b2 are polyepitopic (*11*), and our analyses suggest that CD8^+^ T cell recognition of Omicron S glycoprotein epitopes is largely preserved. Our data show that a third BNT162b2 dose effectively neutralizes Omicron at a similar order of magnitude to that observed for wild-type SARS-CoV-2 after two doses of BNT162b2. Early reports estimate moderate to high vaccine effectiveness against symptomatic Omicron infection, especially shortly after dose three: In the UK, 65 to 75% effectiveness has been reported 2 to 4 weeks after the booster dose, dropping to 55 to 70% at 5 to 9 weeks and below 55% >10 weeks after the third dose (*27*, *28*). Further clinical trial and real-world data will soon emerge to elucidate the effectiveness of a third dose of BNT162b2 against COVID-19 caused by the Omicron variant.

## Supplementary Material

20220118-1

## Supplementary Materials

### This PDF file includes:

Materials and Methods Figs. S1 to S4 Tables S1 to S5 References (*29*–*32*)

### Other Supplementary Material for this manuscript includes the following:

MDAR Reproducibility Checklist

## Notes

View/request a protocol for this paper from *Bio-protocol*.
