# Supplementary material for: Neutralization of SARS-CoV-2 Omicron by BNT162b2 mRNA vaccine–elicited human sera
Source: Science. 2022 Jan 18;375(6581):678–80. doi: 10.1126/science.abn7591 (PMC9836206; doi:10.1126/science.abn7591)
Supplement: 20220118-1 [file science.abn7591.v1.pdf]

Cite as: A. Muik et al., Science 10.1126/science.abn7591 (2022).

Neutralization of SARS-CoV-2 Omicron by BNT162b2 mRNA vaccine-elicited human sera

Alexander Muik[1], Bonny Gaby Lui[1], Ann-Kathrin Wallisch[1], Maren Bacher[1], Julia Mühl[1], Jonas Reinholz[1], Orkun Ozhelvaci[1], Nina Beckmann[1], Ramón de la Caridad Güimil Garcia[1], Asaf Poran[2], Svetlana Shpyro[1], Andrew Finlayson[1], Hui Cai[3], Qi Yang[3], Kena A. Swanson[3], Özlem Türeci[1,4], Uğur Şahin[1,5]*

[1]BioNTech, An der Goldgrube 12, 55131 Mainz, Germany. [2]BioNTech US, 40 Erie Street, Cambridge, MA 02139, USA. [3]Pfizer, 401 N Middletown Rd., Pearl River, NY 10960, USA. [4]HI-TRON – Helmholtz Institute for Translational Oncology Mainz by DKFZ, Obere Zahlbacherstr. 63, 55131 Mainz, Germany. [5]TRON gGmbH – Translational Oncology at the University Medical Center of the Johannes Gutenberg, University Freiligrathstraße 12, 55131 Mainz, Germany.

*Corresponding author. Email: ugur.sahin@biontech.de

The globally-circulating SARS-CoV-2 Variant of Concern Omicron (B.1.1.529) has a large number of mutations especially in the spike protein, indicating that recognition by neutralizing antibodies may be compromised. We tested Wuhan, Beta, Delta, or Omicron pseudoviruses with sera of 51 participants that received two or three doses of the mRNA-based COVID-19 vaccine BNT162b2. Following two doses, sera had >22-fold reduced neutralizing titers against Omicron compared to Wuhan pseudovirus. One month after the third vaccine dose, Omicron-neutralizing titers were increased 23-fold compared to two doses, with titers similar to Wuhan-neutralizing titers after two doses. The requirement of a third vaccine dose to effectively neutralize Omicron was confirmed using live SARS-CoV-2 in a subset of participants. These data suggest that three doses of the mRNA vaccine BNT162b2 may protect against Omicron-mediated COVID-19.

Since the first reports of severe acute respiratory syndrome coronavirus 2 (SARS-CoV-2) in humans in December 2019, numerous genetically distinct lineages have evolved. Among those, variants of concern (VOCs), especially Alpha and Delta variants, were associated with increased viral transmissibility and sparked new waves of infection, with Delta, first designated a VOC on 11th May 2021 (*1*), quickly becoming a globally dominant variant (*2*). On November 26th 2021, a new VOC – Omicron – was reported by the World Health Organization (WHO) (*3*). Omicron is a highly divergent variant and harbors a hitherto unprecedented number of mutations in its spike (S) glycoprotein (*4*). Fifteen mutations are located in the receptor-binding domain (RBD) and another eight mutated sites are found in the N-terminal domain (NTD), both being immunodominant targets of neutralizing antibodies elicited by COVID-19 vaccines or by SARS-CoV-2 infection (*5*, *6*). Some amino acid changes (Δ69/70, T95I, G142D, Δ145, K417N, T478K, N501Y, and P681H) are shared mutations also found in the Alpha, Beta, Gamma or Delta VOCs and were described to lead to increased transmissibility, and to a typically mild partial escape from vaccine-induced humoral immunity (*7*–*10*).

The BNT162b2 COVID-19 mRNA vaccine contains lipid nanoparticle formulated mRNA that encodes the SARS-CoV-2 spike glycoprotein from the parental Wuhan reference strain (*11*). Administration of two 30-μg doses of BNT162b2 was shown to have 95% efficacy in a Phase 3 trial (*12*), and shown to elicit strong antibody responses, effectively neutralizing the parental strain as well as diverse SARS-CoV-2 VOCs (*13*–*15*). As neutralizing antibody titers are strongly predictive with the degree of immune protection against symptomatic SARS-CoV-2 infection (*16*), it is important to understand the effect of the new mutations in Omicron on recognition by neutralizing antibodies in convalescent and vaccinated individuals.

To evaluate whether BNT162b2-elicited antibodies (*11*) are capable of neutralizing the Omicron variant, we used two orthogonal test systems: a pseudovirus neutralization test (pVNT) that has been shown to be in close concordance with live SARS-CoV-2 neutralization assays (*17*, *18*) as well as a live SARS-CoV-2 neutralization test (VNT). For the former, we generated vesicular stomatitis virus (VSV)-SARS-CoV-2-S pseudoviruses bearing the spike proteins of either the Wuhan strain, Omicron, Beta (as a benchmark for partially reduced neutralization (*7*) without major impact on effectiveness (*19*, *20*)) or Delta (the predominant strain until mid-December 2021). BNT162b2 immune sera from vaccinated individuals between 20-72 years of age (with over one third being 56 years of age and older, table S1) were obtained from different clinical trials – the Phase 1/2 trial BNT162-01 (NCT04380701); the Phase 2 rollover trial BNT162-14 (NCT04949490) conducted in Germany; and the global Phase 2 trial BNT162-17 (NCT05004181) (see methods). Neutralizing titers against VSV-SARS-CoV-2-S pseudoviruses were analyzed with serum drawn from 32 participants from the BNT162-01 trial 21 days (median of 22 days; range 19-23 days) after two doses of BNT162b2 (median time from dose 1 to dose 2 was 21 days;

range 19-23 days; table S1), and with serum drawn from 30 participants from the BNT162-14 (n = 11) and BNT162-17 (n = 19) trials at 1 month (median of 28 days; range 26-30 days) after the third dose of BNT162b2 (Median time from dose 2 to dose 3 was 219 days; range 180-342 days). Eleven of the individuals in this analysis were rolled over from the BNT162-01 into the BNT162-14 trial and were included in a longitudinal analysis of neutralizing antibody responses against Wuhan or Omicron variant pseudovirus. These individuals were immunized with a third dose of BNT162b2, with sera collected 1) 21 days (median of 21 days; range 19-23 days) after the second dose, 2) sera collected directly prior to the third dose (median 256 days after dose 2; range 180-342 days), and 3) sera collected at one month (all 28 days) after the third dose.

After two doses of BNT162b2, geometric mean neutralization titers (GMT) against Omicron pseudovirus were 22.8-fold lower compared to the Wuhan reference pseudovirus (Fig. 1; GMT of 7 vs. 160). 20 out of 32 immune sera displayed no detectable neutralizing activity against Omicron (table S2). In contrast, the majority of sera neutralized Beta and Delta pseudoviruses with GMTs of 24 and 73, respectively. This corresponds to a 6.7-fold and 2.2-fold reduction in neutralization activity compared to the Wuhan pseudovirus and is in line with previous reports (*11*, *14*, *15*, *21*).

One month after the third BNT162b2 dose, neutralizing GMTs against the Omicron variant pseudovirus increased 23.4-fold compared to neutralizing GMTs at 21 days after the second dose (GMT of 164 vs. 7); achieving titers comparable to the neutralization against the reference Wuhan pseudovirus at 21 days after two doses of BNT162b2 (GMT of 164 vs. 160). 29 out of 30 sera were capable of neutralizing the Omicron pseudovirus (table S3). The third dose of BNT162b2 also increased neutralizing activity against Beta, Delta and Wuhan pseudoviruses, with GMTs of 279, 413, and 368, respectively.

For 11 individuals that were included in the above analyses a longitudinal analysis of neutralizing titers against Omicron and Wuhan pseudovirus was performed. 21 days after dose 2, sera exhibited a 21.4-fold reduction in GMT against the Omicron variant compared to the Wuhan reference pseudovirus (fig. S1; GMT of 7 vs. 150). Prior to receiving the third dose of BNT162b2 (at a median 256 days following dose 2), neutralizing titers against the Wuhan pseudovirus were considerably reduced (GMT of 13) while the Omicron-specific titers were below the limit of detection. Consistent with the larger serum panel, the third dose of BNT162b2 resulted in a significant increase in neutralizing titers against the Wuhan pseudovirus (GMT of 320) and a 25.8-fold increase in neutralizing titers against Omicron 1 month after dose 3 compared to titers 21 days after dose 2 (GMT of 181 vs. 7).

Sera from a subset of trial participants were analyzed with the second neutralization assay using live SARS-CoV-2 Wuhan and Omicron virus. To this aim, serum from 32 and 25 participants in trial BNT162-01 drawn at 21 days after dose 2, and serum from 7 and 28 participants in the BNT162-14 (n = 7 and n = 11) and BNT162-17 trials (n = 0 and n = 17) drawn at 1 month after dose 3 were tested for neutralization against SARS-CoV-2 Wuhan and Omicron, respectively. Neutralizing GMTs against live SARS-CoV-2 Omicron were 61.3-fold lower compared to the Wuhan reference (Fig. 2; GMT of 6 vs. 368) at 21 days after two doses of BNT162b2. 17 out of 25 immune sera displayed no detectable neutralizing activity against Omicron (table S4). One month after the third BNT162b2 dose, neutralizing GMTs against Omicron increased 17.6-fold compared to neutralizing GMTs at 21 days after the second dose (GMT of 106 vs. 6) and were 3.4-fold lower as compared to the neutralization against the Wuhan reference at 21 days after two doses of BNT162b2 (GMT of 106 vs. 368). 27 of the 28 post dose 3 sera neutralized live SARS-CoV-2 Omicron (table S5).

The observed SARS-CoV-2 neutralizing GMTs correlated positively with the neutralizing GMTs against VSV-SARS-CoV-2-S pseudoviruses (fig. S2).

BNT162b2 vaccination induces strong poly-epitopic T cell responses, directed against multiple epitopes spanning the length of the spike protein (*11*). To assess the risk of immune evasion of $CD8^+$ T cell responses by Omicron, we investigated a set of HLA class I restricted T cell epitopes from the Wuhan spike protein sequence that were reported in the Immune Epitope Database to be immunogenic (IEDB, n = 244; see methods). Despite the multitude of mutations in the Omicron spike protein, 85.3% (n = 208) of the described class I epitopes were not impacted on the amino acid sequence level, indicating that the targets of the vast majority of T cell responses elicited by BNT162b2 may still be conserved in the Omicron variant (fig. S3).

In summary, our data indicate that two doses of the BNT162b2 mRNA vaccine may not be sufficient to protect against infection with the Omicron variant. In both neutralization assay platforms, we observed a substantial reduction in neutralizing activity for immune sera drawn 21 days after the primary 2-dose series of BNT162b2, confirming preliminary reports describing a 20- to 40-fold reduction in titers (*22*, *23*). Both assays also showed that a third dose of BNT162b2 boosts Omicron neutralization capability to robust levels. While in the pseudovirus assay Omicron neutralization titers after three doses reach a level similar to that observed after two doses against the Wuhan pseudovirus, live SARS-CoV-2 Omicron neutralizing GMTs after dose 3 were 3.4-fold lower compared to post-two dose the Wuhan neutralizing GMTs. The observed variability in specific titers and fold differences between non-replicating pseudovirus and replicating live virus neutralization assay platforms as well as different SARS-CoV-2 strains are not unexpected. Importantly, the overall trends are similar and demonstrate that

a third dose of BNT162b2 augments antibody-based immunity against Omicron, in line with previous observations that a third vaccination broadens humoral immune responses against VOCs (*24*).

The analysis presented here has evaluated and compared serum panels from different clinical trials with a limited sample size. BNT162-01 trial participants received the first two doses of BNT162b2 21 days apart (median 21 days; range 19-23 days), with the timing of the third dose not consistent between participants. Recent reports indicate that longer dosing intervals (>42 days) between the first and second dose improve immunogenicity, potentially resulting in a more favorable outcome (*25*). Future analyses will evaluate antibody persistence.

Neutralizing antibodies represent a first layer of adaptive immunity against COVID-19. T cell responses play a vital role as a second layer of defense, in particular in the prevention of severe COVID-19 (*26*). $CD8^+$ T cell responses in individuals vaccinated with BNT162b2 are poly-epitopic (*11*), and our analyses suggest that $CD8^+$ T cell recognition of Omicron spike glycoprotein epitopes are largely preserved. Our data show that a third BNT162b2 dose effectively neutralizes Omicron at a similar order of magnitude as was observed after two doses of BNT162b2 against wild-type SARS-CoV-2. Early reports estimate moderate to high vaccine effectiveness against symptomatic Omicron infection especially shortly after dose 3; 65 to 75% has been reported from the UK at 2 to 4 weeks after the booster dose, dropping to 55 to 70% at 5-9 weeks and below 55% from >10 weeks after the third dose (*27*, *28*). Further clinical trial and real-world data will soon emerge to address the effectiveness of a third dose with BNT162b2 against COVID-19 mediated by Omicron.

REFERENCES AND NOTES

1. World Health Organization, Tracking SARS-CoV-2 variants (available at www.who.int/en/activities/tracking-SARS-CoV-2-variants/).
2. World Health Organization, SARS-CoV-2 Delta variant now dominant in much of European region; efforts must be reinforced to prevent transmission, warns WHO Regional Office for Europe and ECDC (available at www.euro.who.int/en/media-centre/sections/press-releases/2021/sars-cov-2-delta-variant-now-dominant-in-much-of-european-region-efforts-must-be-reinforced-to-prevent-transmission,-warns-who-regional-office-for-europe-and-ecdc).
3. WHO Technical Advisory Group on SARS-CoV-2 Virus Evolution (TAG-VE), Classification of Omicron (B.1.1.529): SARS-CoV-2 Variant of Concern (2021).
4. WHO Headquarters (HQ), WHO Health Emergencies Programme, Enhancing Readiness for Omicron (B.1.1.529): Technical Brief and Priority Actions for Member States (2021).
5. L. Premkumar, B. Segovia-Chumbez, R. Jadi, D. R. Martinez, R. Raut, A. Markmann, C. Cornaby, L. Bartelt, S. Weiss, Y. Park, C. E. Edwards, E. Weimer, E. M. Scherer, N. Rouphael, S. Edupuganti, D. Weiskopf, L. V. Tse, Y. J. Hou, D. Margolis, A. Sette, M. H. Collins, J. Schmitz, R. S. Baric, A. M. de Silva, The receptor binding domain of the viral spike protein is an immunodominant and highly specific target of antibodies in SARS-CoV-2 patients. *Sci. Immunol.* **5**, eabc8413 (2020). doi:10.1126/sciimmunol.abc8413 Medline
6. W. T. Harvey, A. M. Carabelli, B. Jackson, R. K. Gupta, E. C. Thomson, E. M. Harrison, C. Ludden, R. Reeve, A. Rambaut, S. J. Peacock, D. L. Robertson; COVID-19 Genomics UK (COG-UK) Consortium, SARS-CoV-2 variants, spike mutations and immune escape. *Nat. Rev. Microbiol.* **19**, 409–424 (2021). doi:10.1038/s41579-021-00573-0 Medline
7. P. Wang, M. S. Nair, L. Liu, S. Iketani, Y. Luo, Y. Guo, M. Wang, J. Yu, B. Zhang, P. D. Kwong, B. S. Graham, J. R. Mascola, J. Y. Chang, M. T. Yin, M. Sobieszczyk, C. A. Kyratsous, L. Shapiro, Z. Sheng, Y. Huang, D. D. Ho, Antibody resistance of SARS-CoV-2 variants B.1.351 and B.1.1.7. *Nature* **593**, 130–135 (2021). doi:10.1038/s41586-021-03398-2 Medline
8. D. Planas, D. Veyer, A. Baidaliuk, I. Staropoli, F. Guivel-Benhassine, M. M. Rajah, C. Planchais, F. Porrot, N. Robillard, J. Puech, M. Prot, F. Gallais, P. Gantner, A. Velay, J. Le Guen, N. Kassis-Chikhani, D. Edriss, L. Belec, A. Seve, L. Courtellemont, H. Péré, L. Hocqueloux, S. Fafi-Kremer, T. Prazuck, H. Mouquet, T. Bruel, E. Simon-Lorière, F. A. Rey, O. Schwartz, Reduced sensitivity of SARS-CoV-2 variant Delta to antibody neutralization. *Nature* **596**, 276–280 (2021). doi:10.1038/s41586-021-03777-9 Medline
9. Q. Wang, M. S. Nair, S. Anang, S. Zhang, H. Nguyen, Y. Huang, L. Liu, D. D. Ho, J. G. Sodroski, Functional differences among the spike glycoproteins of multiple emerging severe acute respiratory syndrome coronavirus 2 variants of concern. *iScience* **24**, 103393 (2021). doi:10.1016/j.isci.2021.103393 Medline
10. A. J. Greaney, T. N. Starr, P. Gilchuk, S. J. Zost, E. Binshtein, A. N. Loes, S. K. Hilton, J. Huddleston, R. Eguia, K. H. D. Crawford, A. S. Dingens, R. S. Nargi, R. E. Sutton, N. Suryadevara, P. W. Rothlauf, Z. Liu, S. P. J. Whelan, R. H. Carnahan, J. E. Crowe Jr., J. D. Bloom, Complete Mapping of Mutations to the SARS-CoV-2 Spike Receptor-Binding Domain that Escape Antibody Recognition. *Cell Host Microbe* **29**, 44–57.e9 (2021). doi:10.1016/j.chom.2020.11.007 Medline
11. U. Sahin, A. Muik, I. Vogler, E. Derhovanessian, L. M. Kranz, M. Vormehr, J. Quandt, N. Bidmon, A. Ulges, A. Baum, K. E. Pascal, D. Maurus, S. Brachtendorf, V. Lörks, J. Sikorski, P. Koch, R. Hilker, D. Becker, A.-K. Eller, J. Grützner, M. Tonigold, C. Boesler, C. Rosenbaum, L. Heesen, M.-C. Kühnle, A. Poran, J. Z. Dong, U. Luxemburger, A. Kemmer-Brück, D. Langer, M. Bexon, S. Bolte, T. Palanche, A. Schultz, S. Baumann, A. J. Mahiny, G. Boros, J. Reinholz, G. T. Szabó, K. Karikó, P.-Y. Shi, C. Fontes-Garfias, J. L. Perez, M. Cutler, D. Cooper, C. A. Kyratsous, P. R. Dormitzer, K. U. Jansen, Ö. Türeci, BNT162b2 vaccine induces neutralizing antibodies and poly-specific T cells in humans. *Nature* **595**, 572–577 (2021). doi:10.1038/s41586-021-03653-6 Medline
12. F. P. Polack, S. J. Thomas, N. Kitchin, J. Absalon, A. Gurtman, S. Lockhart, J. L. Perez, G. Pérez Marc, E. D. Moreira, C. Zerbini, R. Bailey, K. A. Swanson, S. Roychoudhury, K. Koury, P. Li, W. V. Kalina, D. Cooper, R. W. Frenck Jr., L. L. Hammitt, Ö. Türeci, H. Nell, A. Schaefer, S. Ünal, D. B. Tresnan, S. Mather, P. R. Dormitzer, U. Şahin, K. U. Jansen, W. C. Gruber; C4591001 Clinical Trial Group, Safety and Efficacy of the BNT162b2 mRNA Covid-19 Vaccine. *N. Engl. J. Med.* **383**, 2603–2615 (2020). doi:10.1056/NEJMoa2034577 Medline
13. A. Muik, A.-K. Wallisch, B. Sänger, K. A. Swanson, J. Mühl, W. Chen, H. Cai, D. Maurus, R. Sarkar, Ö. Türeci, P. R. Dormitzer, U. Şahin, Neutralization of SARS-CoV-2 lineage B.1.1.7 pseudovirus by BNT162b2 vaccine-elicited human sera. *Science* **371**, 1152–1153 (2021). doi:10.1126/science.abg6105 Medline
14. J. Liu, Y. Liu, H. Xia, J. Zou, S. C. Weaver, K. A. Swanson, H. Cai, M. Cutler, D. Cooper, A. Muik, K. U. Jansen, U. Sahin, X. Xie, P. R. Dormitzer, P.-Y. Shi, BNT162b2-elicited neutralization of B.1.617 and other SARS-CoV-2 variants. *Nature* **596**, 273–275 (2021). doi:10.1038/s41586-021-03693-y Medline
15. Y. Liu, J. Liu, H. Xia, X. Zhang, C. R. Fontes-Garfias, K. A. Swanson, H. Cai, R. Sarkar, W. Chen, M. Cutler, D. Cooper, S. C. Weaver, A. Muik, U. Sahin, K. U. Jansen, X. Xie, P. R. Dormitzer, P.-Y. Shi, Neutralizing Activity of BNT162b2-Elicited Serum. *N. Engl. J. Med.* **384**, 1466–1468 (2021). doi:10.1056/NEJMc2102017 Medline
16. D. S. Khoury, D. Cromer, A. Reynaldi, T. E. Schlub, A. K. Wheatley, J. A. Juno, K. Subbarao, S. J. Kent, J. A. Triccas, M. P. Davenport, Neutralizing antibody levels are highly predictive of immune protection from symptomatic SARS-CoV-2 infection. *Nat. Med.* **27**, 1205–1211 (2021). doi:10.1038/s41591-021-01377-8 Medline
17. J. B. Case, P. W. Rothlauf, R. E. Chen, Z. Liu, H. Zhao, A. S. Kim, L.-M. Bloyet, Q. Zeng, S. Tahan, L. Droit, M. X. G. Ilagan, M. A. Tartell, G. Amarasinghe, J. P. Henderson, S. Miersch, M. Ustav, S. Sidhu, H. W. Virgin, D. Wang, S. Ding, D. Corti, E. S. Theel, D. H. Fremont, M. S. Diamond, S. P. J. Whelan, Neutralizing Antibody and Soluble ACE2 Inhibition of a Replication-Competent VSV-SARS-CoV-2 and a Clinical Isolate of SARS-CoV-2. *Cell Host Microbe* **28**, 475–485.e5 (2020). doi:10.1016/j.chom.2020.06.021 Medline
18. A. B. Vogel, I. Kanevsky, Y. Che, K. A. Swanson, A. Muik, M. Vormehr, L. M. Kranz, K. C. Walzer, S. Hein, A. Güler, J. Loschko, M. S. Maddur, A. Ota-Setlik, K.

Tompkins, J. Cole, B. G. Lui, T. Ziegenhals, A. Plaschke, D. Eisel, S. C. Dany, S. Fesser, S. Erbar, F. Bates, D. Schneider, B. Jesionek, B. Sänger, A.-K. Wallisch, Y. Feuchter, H. Junginger, S. A. Krumm, A. P. Heinen, P. Adams-Quack, J. Schlereth, S. Schille, C. Kröner, R. de la Caridad Güimil Garcia, T. Hiller, L. Fischer, R. S. Sellers, S. Choudhary, O. Gonzalez, F. Vascotto, M. R. Gutman, J. A. Fontenot, S. Hall-Ursone, K. Brasky, M. C. Griffor, S. Han, A. A. H. Su, J. A. Lees, N. L. Nedoma, E. H. Mashalidis, P. V. Sahasrabudhe, C. Y. Tan, D. Pavliakova, G. Singh, C. Fontes-Garfias, M. Pride, I. L. Scully, T. Ciolino, J. Obregon, M. Gazi, R. Carrion Jr., K. J. Alfson, W. V. Kalina, D. Kaushal, P.-Y. Shi, T. Klamp, C. Rosenbaum, A. N. Kuhn, Ö. Türeci, P. R. Dormitzer, K. U. Jansen, U. Sahin, BNT162b vaccines protect rhesus macaques from SARS-CoV-2. *Nature* **592**, 283–289 (2021). doi:10.1038/s41586-021-03275-y Medline

19. L. J. Abu-Raddad, H. Chemaitelly, A. A. Butt; National Study Group for COVID-19 Vaccination, Effectiveness of the BNT162b2 Covid-19 Vaccine against the B.1.1.7 and B.1.351 Variants. *N. Engl. J. Med.* **385**, 187–189 (2021). doi:10.1056/NEJMc2104974 Medline
20. O. Mor, N. S. Zuckerman, I. Hazan, R. Fluss, N. Ash, N. Ginish, E. Mendelson, S. Alroy-Preis, L. Freedman, A. Huppert, BNT162b2 vaccine effectiveness was marginally affected by the SARS-CoV-2 beta variant in fully vaccinated individuals. *J. Clin. Epidemiol.* **142**, 38–44 (2022). doi:10.1016/j.jclinepi.2021.10.011 Medline
21. A. Kuzmina, S. Wattad, Y. Khalaila, A. Ottolenghi, B. Rosental, S. Engel, E. Rosenberg, R. Taube, SARS CoV-2 Delta variant exhibits enhanced infectivity and a minor decrease in neutralization sensitivity to convalescent or post-vaccination sera. *iScience* **24**, 103467 (2021). doi:10.1016/j.isci.2021.103467 Medline
22. S. Cele, L. Jackson, D. S. Khoury, K. Khan, T. Moyo-Gwete, H. Tegally, J. E. San, D. Cromer, C. Scheepers, D. Amoako, F. Karim, M. Bernstein, G. Lustig, D. Archary, M. Smith, Y. Ganga, Z. Jule, K. Reedoy, S.-H. Hwa, J. Giandhari, J. M. Blackburn, B. I. Gosnell, S. S. Abdool Karim, W. Hanekom, NGS-SA, COMMIT-KZN Team, A. von Gottberg, J. Bhiman, R. J. Lessells, M.-Y. S. Moosa, M. P. Davenport, T. de Oliveira, P. L. Moore, A. Sigal, SARS-CoV-2 Omicron has extensive but incomplete escape of Pfizer BNT162b2 elicited neutralization and requires ACE2 for infection. medRxiv 2021.12.08.21267417 (2021); https://doi.org/10.1101/2021.12.08.21267417.
23. A. Wilhelm, M. Widera, K. Grikscheit, T. Toptan, B. Schenk, C. Pallas, M. Metzler, N. Kohmer, S. Hoehl, F. A. Helfritz, T. Wolf, U. Goetsch, S. Ciesek, Reduced Neutralization of SARS-CoV-2 Omicron Variant by Vaccine Sera and Monoclonal Antibodies. medRxiv 2021.12.07.21267432 (2021); https://doi.org/10.1101/2021.12.07.21267432.
24. A. R. Falsey, R. W. Frenck Jr., E. E. Walsh, N. Kitchin, J. Absalon, A. Gurtman, S. Lockhart, R. Bailey, K. A. Swanson, X. Xu, K. Koury, W. Kalina, D. Cooper, J. Zou, X. Xie, H. Xia, Ö. Türeci, E. Lagkadinou, K. R. Tompkins, P.-Y. Shi, K. U. Jansen, U. Şahin, P. R. Dormitzer, W. C. Gruber, SARS-CoV-2 Neutralization with BNT162b2 Vaccine Dose 3. *N. Engl. J. Med.* **385**, 1627–1629 (2021). doi:10.1056/NEJMc2113468 Medline
25. B. Grunau, D. M. Goldfarb, M. Asamoah-Boaheng, L. Golding, T. L. Kirkham, P. A. Demers, P. M. Lavoie, Immunogenicity of Extended mRNA SARS-CoV-2 Vaccine Dosing Intervals. *JAMA* (2021). doi:10.1001/jama.2021.21921 Medline
26. A. Bertoletti, N. Le Bert, M. Qui, A. T. Tan, SARS-CoV-2-specific T cells in infection and vaccination. *Cell. Mol. Immunol.* **18**, 2307–2312 (2021). doi:10.1038/s41423-021-00743-3 Medline
27. UK Health Security Agency, SARS-CoV-2 variants of concern and variants under investigation in England - Technical briefing: Update on hospitalisation and vaccine effectiveness for Omicron VOC-21NOV-01 (B.1.1.529) (available at https://assets.publishing.service.gov.uk/government/uploads/system/uploads/attachment_data/file/1044481/Technical-Briefing-31-Dec-2021-Omicron_severity_update.pdf).
28. N. Ferguson *et al*., Report 49: Growth, population distribution and immune escape of Omicron in England (2021) (available at www.imperial.ac.uk/media/imperial-college/medicine/mrc-gida/2021-12-16-COVID19-Report-49.pdf).
29. S. Khare, C. Gurry, L. Freitas, M. B. Schultz, G. Bach, A. Diallo, N. Akite, J. Ho, R. T. Lee, W. Yeo, GISAID Core Curation Team, S. Maurer-Stroh, GISAID's Role in Pandemic Response. *China CDC Weekly* **3**, 1049–1051 (2021). doi:10.46234/ccdcw2021.255 Medline
30. K. Katoh, D. M. Standley, MAFFT multiple sequence alignment software version 7: Improvements in performance and usability. *Mol. Biol. Evol.* **30**, 772–780 (2013). doi:10.1093/molbev/mst010 Medline
31. M. Berger Rentsch, G. Zimmer, A vesicular stomatitis virus replicon-based bioassay for the rapid and sensitive determination of multi-species type I interferon. *PLOS ONE* **6**, e25858 (2011). doi:10.1371/journal.pone.0025858 Medline
32. W. Fleri, N. Salimi, R. Vita, B. Peters, A. Sette, Immune Epitope Database and Analysis Resource (2016). *Encyclopedia of Immunobiology*. **2** (2016).

ACKNOWLEDGMENTS

We thank the BioNTech German clinical Phase 1/2 trial (NCT04380701, EudraCT: 2020-001038-36), the German Phase 2 rollover booster trial (NCT04949490, EudraCT: 2021-002387-50) and the global clinical Phase 2 trial (NCT04380701) participants, from whom the post-immunization human sera were obtained. We thank the many colleagues at BioNTech and Pfizer who developed and produced the BNT162b2 mRNA vaccine candidate. We thank S. Jägle for logistical support, and B. Huang for support generating the overview figure S4. We thank the VisMederi team for performing excellent work on live virus-neutralizing antibody assays. We thank C. Heiser, A. Telorman, K. Krüger, C. Müller, A. Wanamaker, N. Williams and J. VanCamp for sample demographics support. **Funding:** This work was supported by BioNTech and Pfizer. **Author contributions:** U.S., Ö.T., and A.M. conceived and conceptualized the work. A.M, B.G.L., J.R., H.C., Q.Y., K.A.S and R.G planned and supervised experiments. A.M., A.W., B.G.L, J.M., J.R., M.B., N.B. and R.G. performed experiments. A.M., A.P., B.G.L., J.R., K.A.S., O.O., R.G., and S.S. and analyzed data. U.S., Ö.T., A.M., A.F., and K.A.S. interpreted data and wrote the manuscript. All authors supported the review of the manuscript. **Competing interests:** U.S. and Ö.T. are management board members and employees at BioNTech SE. A.F., A.M., A.W., B.G.L, J.M., J.R., M.B, N.B., O.O., S.S. and R.G. are employees at BioNTech SE. A.P. is an employee at BioNTech US. U.S., Ö.T. and A.M. are inventors on patents and patent applications related to RNA technology and COVID-19 vaccine. U.S., Ö.T., N.B., A.F., A.M., A.P., A.W., B.G.L., J.M., O.O., J.R., S.S., and R.G. have securities from BioNTech SE; H.C., Q.Y., K.A.S. are employees at Pfizer and may have securities from Pfizer. **Data and materials availability:** All data are available in the main manuscript or the supplementary materials. Trial participant baseline characteristics are provided in table S1. The neutralization titers are provided in tables S2 to S5. Materials are available from the authors under a material transfer agreement with BioNTech. This work is licensed under a Creative Commons Attribution 4.0 International (CC BY 4.0) license, which permits unrestricted use, distribution, and reproduction in any medium, provided the original work is properly cited. To view a copy of this license, visit https://creativecommons.org/licenses/by/4.0/. This license does not apply to figures/photos/artwork or other content included in the article that is credited to a third party; obtain authorization from the rights holder before using such material.

SUPPLEMENTARY MATERIALS

science.org/doi/10.1126/science.abn7591
Materials and Methods
Figs. S1 to S4
Tables S1 to S5
References (*29*–*32*)
MDAR Reproducibility Checklist

20 December 2021; accepted 11 January 2022
Published online 18 January 2022
10.1126/science.abn7591

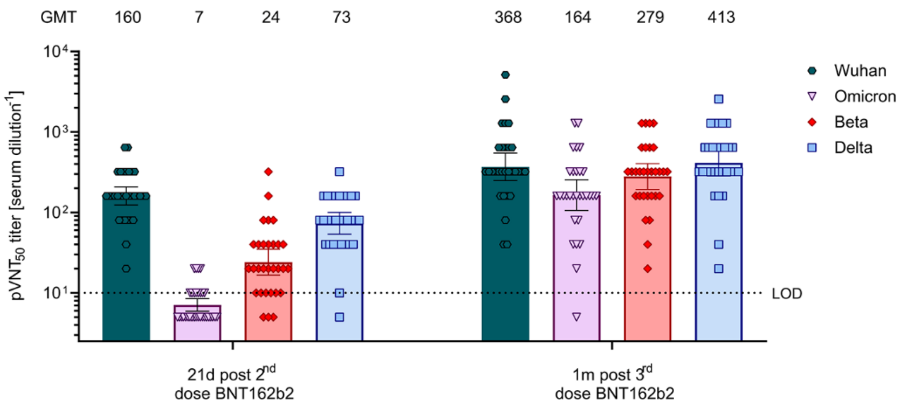

Fig. 1. 50% pseudovirus neutralization titers ($pVNT_{50}$) of sera from vaccine recipients collected after two or three doses of BNT162b2 against VSV-SARS-CoV-2-S pseudovirus bearing the Wuhan, Omicron, Beta or Delta variant spike protein. N = 32 sera from participants in trial BNT162-01 drawn at 21 days after dose 2, and n = 30 sera from participants in the BNT162-14 (n = 11) and BNT162-17 trials (n = 19) drawn at 1 month after dose 3 were tested. Each serum was tested in duplicate and geometric mean 50% pseudovirus neutralizing titers (GMTs) were plotted. For values below the limit of detection (LOD), LOD/2 values were plotted. Group GMTs (values) and 95% confidence intervals are indicated.

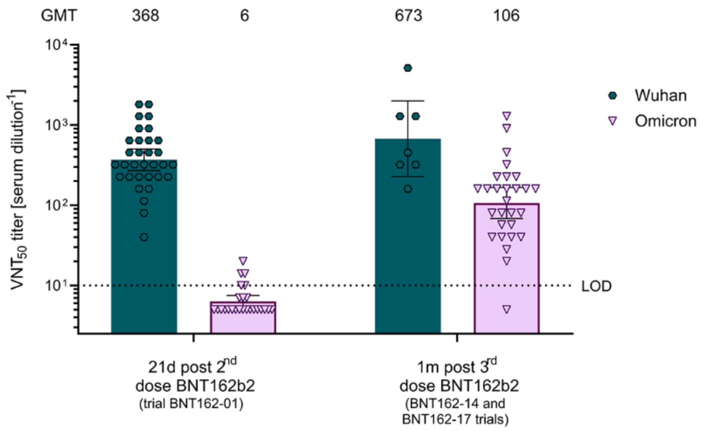

Fig. 2. 50% live SARS-CoV-2 neutralization titers (VNT_{50}) of sera from vaccine recipients collected after two or three doses of BNT162b2. Sera from participants in trial BNT162-01 drawn at 21 days after dose 2 were tested for neutralization against SARS-CoV-2 Wuhan (n = 32) and Omicron (n = 25), respectively. Sera from participants in the BNT162-14 (n = 7 and n = 11) and BNT162-17 trials (n = 0 and n = 17) drawn at 1 month after dose 3 were tested for neutralization against SARS-CoV-2 Wuhan (total n = 7) and Omicron (total n = 28), respectively. Each serum was tested in duplicate and geometric mean 50% SARS-CoV-2 neutralizing titers (GMTs) were plotted. For values below the limit of detection (LOD), LOD/2 values were plotted. Group GMTs (values) and 95% confidence intervals are indicated.
