# Supplementary material for: Neutralization of SARS-CoV-2 Omicron by BNT162b2 mRNA vaccine–elicited human sera
Source: Science. 2022 Jan 18;375(6581):678–80. doi: 10.1126/science.abn7591 (PMC9836206; doi:10.1126/science.abn7591)
Supplement: Supplementary file 2 — Materials and Methods Figs. S1 to S4 Tables S1 to S5 References (29–32) [file science.abn7591_sm.pdf]

Science
AAAS

Supplementary Materials for

Neutralization of SARS-CoV-2 Omicron by BNT162b2 mRNA vaccine–elicited human sera

Alexander Muik *et al*.

Corresponding author: Uğur Şahin, ugur.sahin@biontech.de

Science **375**, 678 (2022)
DOI: 10.1126/science.abn7591

The PDF file includes:

Materials and Methods
Figs. S1 to S4
Tables S1 to S5
References

Other Supplementary Material for this manuscript includes the following:

MDAR Reproducibility Checklist

Materials and Methods

Clinical Trials

This research used samples from 18-85 year old participants of the German Phase 1/2 trial BNT162-01 (NCT04380701) vaccinated with 2-dose primary series of 30 µg BNT162b2 with a 21 ± 2 days dosing interval, that has been described elsewhere (*11*). In addition, samples were used from participants in subgroups of two ongoing clinical trials, BNT162-14 (NCT04949490) aged 18-85 and BNT162-17 (NCT05004181) aged 18-55 years old.

Participants of BNT162-01 who received the 2-dose primary series with BNT162b2 were recruited to the BNT162-14 clinical trial, a Phase 2, open-label, rollover trial located at multiple sites in Germany. Participants included here were vaccinated with a booster dose of BNT162b2 at least 6 months and less than 18 months since dose 2 in the parental BNT162-01 trial.

BNT162-17 is an ongoing Phase 2 clinical trial located at multiple sites in the US, Germany, Turkey and South Africa. Trial participants were vaccinated with BNT162b2 vaccine (30 µg, two-dose primary series) in either a clinical trial or as part of the governmental vaccination programs at least 6 months before receiving dose 3.

Participants included in this study from these clinical trials were from subcohorts contributing to the exploratory endpoint to evaluate cross-neutralization of BNT162b2-induced antibodies to emerging SARS-CoV-2 variants following 2-dose primary series vaccination with or without booster vaccination (dose 3) in healthy adults.

The trials were carried out in accordance with the Declaration of Helsinki and Good Clinical Practice Guidelines and with approval by independent ethics committees and the competent

regulatory authorities. All participants provided written informed consent. The primary objectives of these trials will be reported at a later date.

Serum specimens

Three different panels of sera were investigated. The first serum panel was obtained from 32 participants in the BNT162-01 trial drawn at a median 22 days (range 19-23 days) after receiving the second dose BNT162b2 of the 2-dose primary series (2 x 30µg). Median age was 57 years (range 20-72 years), 18 participants were ≥56 years old and none had evidence of current or prior SARS-CoV-2 at baseline. Median time from dose 1 to dose 2 was 21 days (range 19-23 days) (Table S1). The second serum panel was obtained from 30 participants in the BNT162-14 (n=11) and BNT162-17 (n=19) trial drawn at a median 28 days (range 26-30 days) after receiving the third dose BNT162b2. Median age was 44 years (range 23-72 years), 8 participants were ≥56 years old and none had evidence of current or prior SARS-CoV-2 at baseline. Median time from dose 2 to dose 3 was 219 days (range 180-342 days). The third serum panel was obtained from 11 participants immunized with a third dose of BNT162b2 in the BNT162-14 trial, who rolled over from the parental trial BNT162-01. For longitudinal analysis serum was collected at a median 21 days (range 19-23 days) after receiving the second dose BNT162b2 in the BNT162-01 trial, at a median 256 days (range 180-342 days) after receiving the second dose BNT162b2 in the BNT162-01 trial directly prior to dose 3 in the BNT162-14 trial, and at 1 month (all 28 days) after receiving the third dose BNT162b2 in the BNT162-14 trial. Median age was 63 years (range 25-72 years), 8 participants were ≥56 years old and none had evidence of current or prior SARS-CoV-2 at baseline. Median time from dose 1 to dose 2 was 21 days (range 19-23 days) and median time from dose 2 to dose 3 was 256 days (range 180-342 days).

Mutation identification for Omicron

All available genome sequences assigned to the B.1.1.529 SARS-CoV-2 lineage were obtained from GISAID (*29*) on November 26th, 2021 (n=77). To retrieve the spike glycoprotein sequence from each sample, the nucleotide sequences were in-silico translated; due to ambiguity in the reading frame, each sample was translated in all three possible reading frames, and the resulting amino acid sequence was searched for the spike beginning (MFVFLVLLP) and ending (GVKLHYT). For each sample, the sequences were found in only one open reading frame. The full spike sequences were aligned using MAFFT (v7.475) (*30*) alongside with the Wuhan strain spike sequence (NCBI Reference Sequence: NC_045512.2). Two sequences were excluded due to poor alignment to the Wuhan strain. Conservation across the B.1.1.529 was used to identify the amino acid changes compared to the Wuhan strain.

VSV-SARS-CoV-2 S variant pseudovirus generation

A recombinant replication-deficient vesicular stomatitis virus (VSV) vector that encodes green fluorescent protein (GFP) and luciferase instead of the VSV-glycoprotein (VSV-G) was pseudotyped with SARS-CoV-2 spike (S) derived from either the Wuhan reference strain (NCBI Ref: 43740568), the Beta variant (mutations: L18F, D80A, D215G, Δ242–244, R246I, K417N, E484K, N501Y, D614G, A701V), the Delta variant (mutations: T19R, G142D, E156G, Δ157/158, K417N, L452R, T478K, D614G, P681R, D950N) or the Omicron variant (mutations: A67V, Δ69/70, T95I, G142D, Δ143-145, Δ211, L212I, ins214EPE, G339D, S371L, S373P, S375F, K417N, N440K, G446S, S477N, T478K, E484A, Q493R, G496S, Q498R, N501Y, Y505H, T547K, D614G, H655Y, N679K, P681H, N764K, D796Y, N856K, Q954H, N969K,

L981F) according to published pseudotyping protocols (*13*, *31*). A diagram of spike mutations is shown in fig. S4A. In brief, HEK293T/17 monolayers (ATCC® CRL-11268™) cultured in Dulbecco's modified Eagle's medium (DMEM) with GlutaMAX™ (Gibco) supplemented with 10% heat-inactivated fetal bovine serum (FBS [Sigma-Aldrich]) (referred to as medium) were transfected with Sanger sequencing-verified variant-specific SARS-CoV-2 S expression plasmid with Lipofectamine LTX (Life Technologies) following the manufacturer's protocol. At 24 hours VSV-G complemented VSVΔG vector. After incubation for 2 hours at 37 °C with 7.5% CO_2, the inoculum was removed. Cells were washed twice with phosphate buffered saline (PBS) before medium supplemented with anti-VSV-G antibody (clone 8G5F11, Kerafast Inc.) was added to neutralize residual VSV-G complemented input virus. VSV-SARS-CoV-2-S pseudotype-containing medium was harvested 20 hours after inoculation, passed through a 0.2 µm filter (Nalgene) and stored at -80 °C. Prior to use in the neutralization test, the pseudovirus batches were titrated on Vero 76 cells (ATCC® CRL-1587™) cultured in medium. The relative luciferase units induced by a defined volume of a Wuhan spike pseudovirus reference batch previously described in Muik et al., 2021 (*13*), that corresponds to an infectious titer of 200 transducing units (TU) per mL, was used as a comparator. Input volumes for the SARS-CoV-2 variant pseudovirus batches were calculated to normalize the infectious titer based on the relative luciferase units relative to the reference. All pseudovirus batches used in this study were titrated simultaneously.

Pseudovirus neutralization assay

Vero 76 cells were seeded in 96-well white, flat-bottom plates (Thermo Scientific) at 40,000 cells/well in medium 4 hours prior to the assay and cultured at 37 °C with 7.5% CO_2. Each serum was 2-fold serially diluted in medium with the first dilution of 1:5 (dilution range of 1:5 to 1:5,120). VSV-SARS-CoV-2-S particles were diluted in medium to obtain 200 TU in the assay. Serum dilutions were mixed 1:1 with pseudovirus (n=2 technical replicates per serum per pseudovirus) for 30 minutes at room temperature prior to addition to Vero 76 cell monolayers and incubation at 37 °C with 7.5% CO_2 for 24 hours. Supernatants were removed, and the cells were lysed with luciferase reagent (Promega). Luminescence was recorded on a CLARIOstar® Plus microplate reader (BMG Labtech), and neutralization titers were calculated as the reciprocal of the highest serum dilution that still resulted in 50% reduction in luminescence. Results were reported as geometric mean titer (GMT) of duplicates. If no neutralization was observed, an arbitrary titer value of 5 (half of the limit of detection [LOD]) was reported. The full set of sera was tested for neutralization of SARS-CoV-2 pseudoviruses in three independent assays. Tables of the neutralization titers are provided (Table S2 and Table S3).

Live SARS-CoV-2 neutralization assay

SARS-CoV-2 virus neutralization titers were determined by a microneutralization assay based on cytopathic effect (CPE) at VisMederi S.r.l., Siena, Italy. In brief, heat-inactivated serum samples from participants were serially diluted 1:2 (starting at 1:10) and incubated for 1 hour at 37 °C with 100 $TCID_{50}$ of live SARS-CoV-2 virus strain 2019-nCOV/ITALY-INMI1 or sequence-verified Omicron strain hCoV-19/Belgium/rega-20174/2021 (mutations: A67V, Δ69/70, T95I, G142D, Δ143-145, Δ211, L212I, ins214EPE, G339D, S371L, S373P, S375F, K417N, N440K,

G446S, S477N, T478K, E484A, Q493R, G496S, Q498R, N501Y, Y505H, T547K, D614G, H655Y, N679K, P681H, N764K, D796Y, N856K, Q954H, N969K, L981F; a diagram of spike mutations is shown in fig. S4B) to allow any antigen-specific antibodies to bind to the virus. The 2019-nCOV/ITALY-INMI1 strain S is identical in sequence to the wild-type SARS-CoV-2 S (Wuhan-Hu-1 isolate). Vero E6 (ATCC® CRL-1586™) cell monolayers were inoculated with the serum/virus mix in 96-well plates and incubated for 3 days (2019-nCOV/ITALY-INMI1 strain) or 4 days (Omicron variant) to allow infection by non-neutralized virus. The plates were observed under an inverted light microscope and the wells were scored as positive for SARS-CoV-2 infection (i.e., showing CPE) or negative for SARS-CoV-2 infection (i.e., cells were alive without CPE). The neutralization titer was determined as the reciprocal of the highest serum dilution that protected more than 50% of cells from CPE and reported as GMT of duplicates. If no neutralization was observed, an arbitrary titer value of 5 (half of the limit of detection [LOD]) was reported. Tables of the neutralization titers are provided (Table S4 and Table S5).

T cell epitope conservation in the Omicron Spike variant

To estimate the rate of nonsynonymous mutation in T cell epitopes in the spike glycoprotein, we used the Immune Epitope Database (https://www.iedb.org/) (*32*) to obtain epitopes confirmed for T cell reactivity in experimental assays. The database was filtered using the following criteria: Organism: SARS-COV2; Antigen: Spike glycoprotein; Positive Assay; No B cell assays; No MHC assays; MHC Restriction Type: Class I; Host: Homo sapiens (human). The resulting table was filtered by removing epitopes that were “deduced from a reactive overlapping peptide pool”, as well as epitopes longer than 14 amino acids in order to restrict the dataset to confirmed minimal epitopes only. The experimental assays confirming the reactivity of these epitopes relied

on multimer analysis, ELISpot or ELISpot-like assays, T cell activation assays, etc. The epitopes were reported for at least 27 different HLA-I alleles, including HLA-A, HLA-B, and HLA-C alleles. Of the 251 unique epitope sequences obtained in this approach, 244 were found in the Wuhan strain spike glycoprotein. Of these, 36 epitopes (14.8%) included a position reported to be mutated by our sequence analysis.

Statistical analysis

The statistical method of aggregation used for the analysis of antibody titers is the geometric mean and the corresponding 95% confidence interval. Using the geometric mean accounts for non-normal distribution of antibody titers that span several orders of magnitude. Spearman correlation was used to evaluate the monotonic relationship between non-normally distributed datasets. All statistical analyses were performed using GraphPad Prism software version 9.

References

11. U. Sahin *et al.,* BNT162b2 vaccine induces neutralizing antibodies and poly-specific T cells in humans. *Nature*. **595**, 572–577 (2021), doi:10.1038/s41586-021-03653-6.
13. A. Muik *et al.,* Neutralization of SARS-CoV-2 lineage B.1.1.7 pseudovirus by BNT162b2 vaccine-elicited human sera. *Science (New York, N.Y.)*. **371**, 1152–1153 (2021), doi:10.1126/science.abg6105.
29. S. Khare *et al.,* GISAID's Role in Pandemic Response. *China CDC Weekly*. **3**, 1049–1051 (2021), doi:10.46234/ccdcw2021.255.
30. K. Katoh, D. M. Standley, MAFFT multiple sequence alignment software version 7. *Molecular biology and evolution*. **30**, 772–780 (2013), doi:10.1093/molbev/mst010.
31. M. Berger Rentsch, G. Zimmer, A vesicular stomatitis virus replicon-based bioassay for the rapid and sensitive determination of multi-species type I interferon. *PloS one*. **6**, e25858 (2011), doi:10.1371/journal.pone.0025858.
32. W. Fleri, N. Salimi, R. Vita, B. Peters, A. Sette, Immune Epitope Database and Analysis Resource (2016). *Encyclopedia of Immunobiology*. **2** (2016).

(The full reference list appears at the end of this document.)

Fig. S1

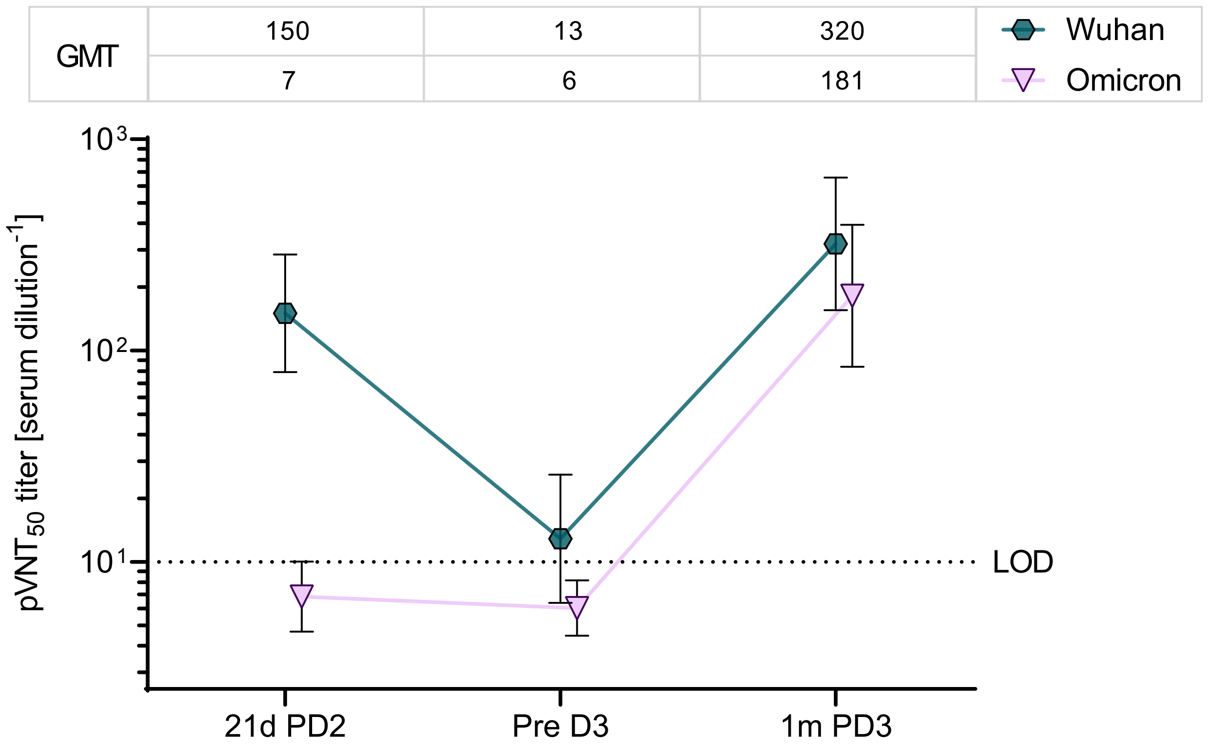

Fig. S1. Longitudinal analysis of neutralizing antibody titers against VSV-SARS-CoV-2-S pseudovirus bearing the Wuhan or Omicron variant spike protein. Sera from n=11 participants in trial BNT162-14 who rolled over from the parental trial BNT162-01 drawn at 21 days after dose 2, prior to dose 3 (at a median 256 days following dose 2) and 1 month after dose 3 were tested. Each serum was tested in duplicate and individual geometric mean 50% pseudovirus neutralizing titers (GMTs) were calculated. For values below the limit of detection (LOD), LOD/2 values were assigned. Group GMTs (values in table) and 95% confidence intervals per time-point are indicated.

LOD, limit of detection; PD, post-dose; d, day; m, month.

Fig. S2

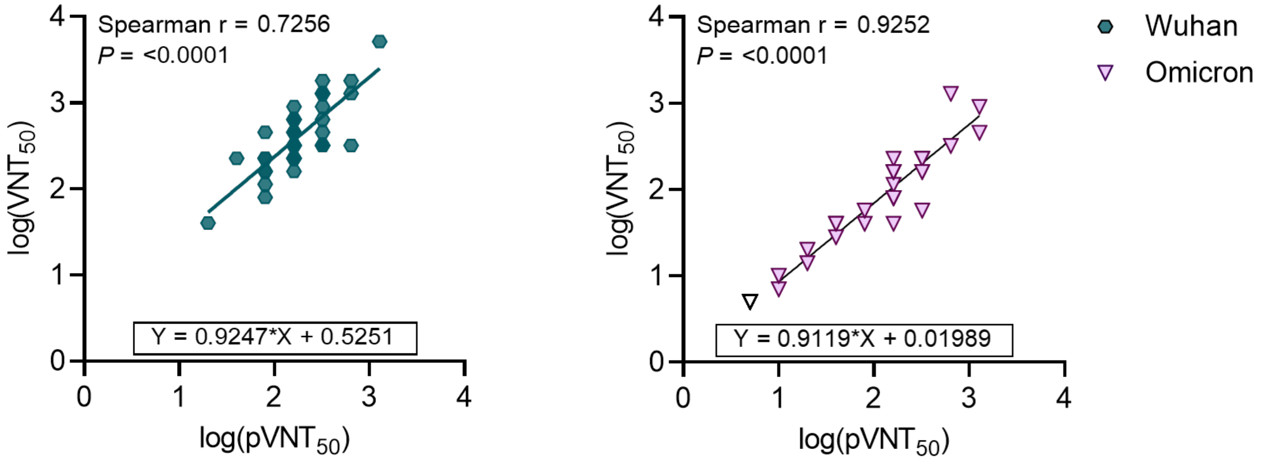

Fig. S2. Non-parametric Spearman correlation of VSV-SARS-CoV-2-S pVNT$_{50}$ with live SARS-CoV-2 VNT$_{50}$ for Wuhan reference and Omicron variant virus. Wuhan neutralization titer data is shown for n=39 serum samples (n=32 drawn at 21 days after dose 2 and n=7 drawn at 1 month after dose 3 of BNT162b2). Omicron neutralization titer data is shown for n=53 serum samples (n=25 drawn at 21 days after dose 2 and n=28 drawn at 1 month after dose 3 of BNT162b2). Data points for serum samples with neutralizing GMTs below the LOD (open triangles) were excluded from the correlation analysis.

Fig. S3

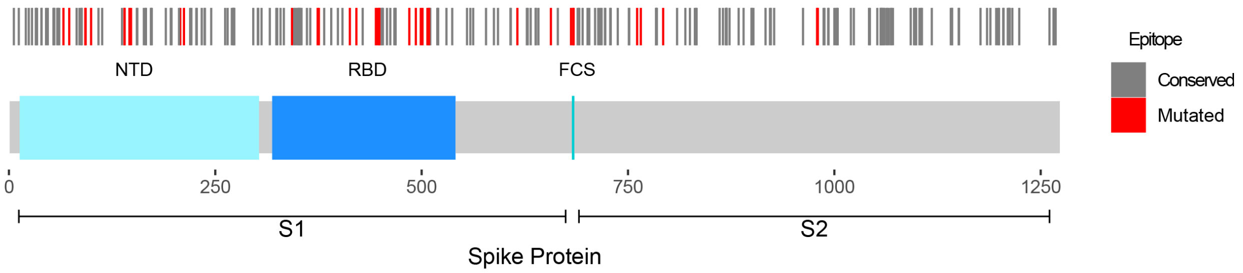

Fig. S3. Conservation of HLA class I T cell epitopes between the Wuhan and Omicron variants. HLA class I restricted spike protein epitopes identified based on their recognition by CD8+ T cells and reported in IEDB (n=244) are plotted by their position (top row) along the Spike protein (bottom row). Epitope indications are positioned by the amino acid position of the center of the epitope; epitopes conserved in both variants are marked in grey (n=208); epitopes spanning an Omicron mutation site are marked red (n=36). NTD, N-terminal domain; RBD, Receptor-binding domain; FCS, Furin cleavage site.

Fig. S4

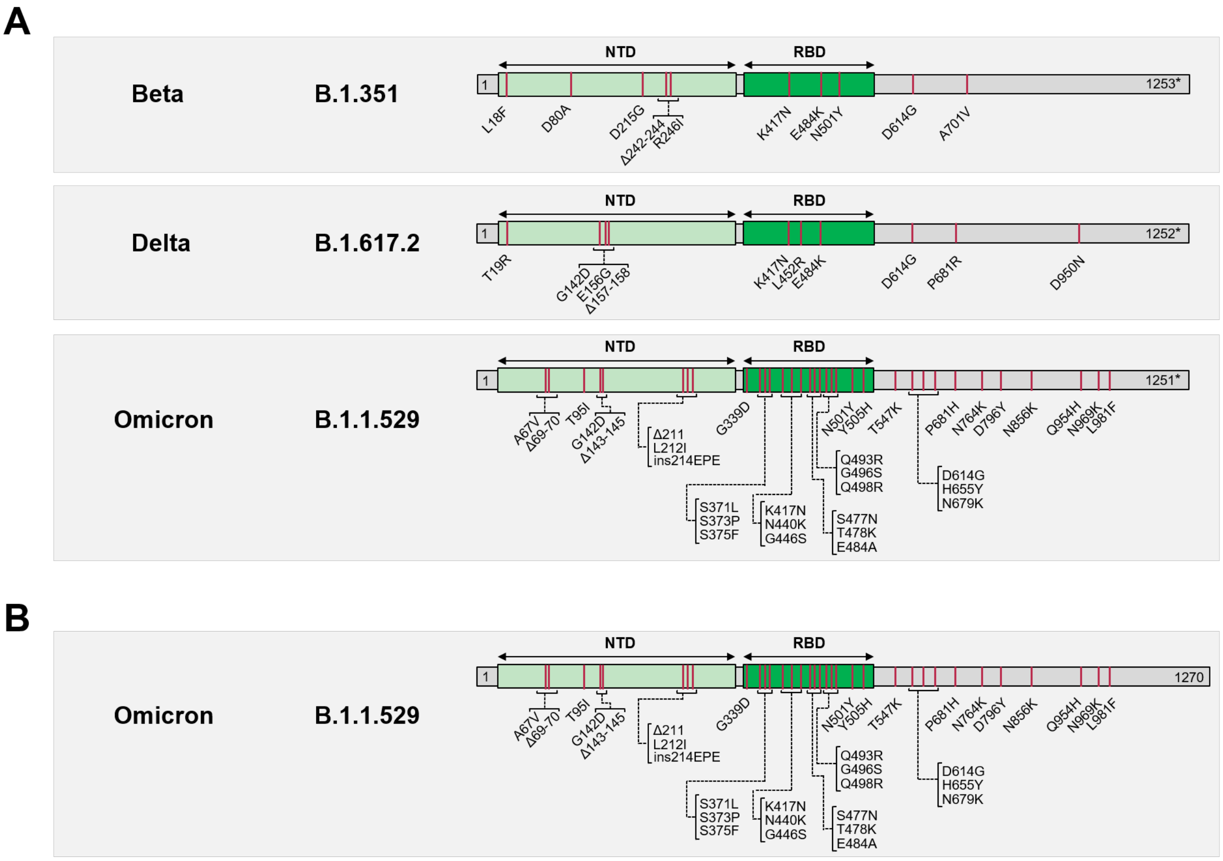

Fig. S4. Characteristics of spike proteins used in the assays based on (a) VSV-SARS-CoV-2 variant pseudoviruses and (b) live SARS-CoV-2 Omicron. The sequence of the Wuhan-Hu-1 isolate SARS-CoV-2 Spike (GenBank: QHD43416.1) was used as reference. Amino acid positions, amino acid descriptions (one letter code) and kind of mutations (substitutions, deletions, insertions) are indicated. NTD, N-terminal domain; RBD, Receptor-binding domain, Δ, deletion; ins, insertion; *, Cytoplasmic domain truncated for the C-terminal 19 amino acids.

Table S1. Cohort characteristics

Characteristic	21d after dose 2 cohort (n=32)	1m after dose 3 cohort (n=30)	Longitudinal analysis cohort (n=11)	All participants (n=51)
Sex, n (%)				
Male	17 (53)	15 (50)	5 (45)	27 (53)
Female	15 (47)	15 (50)	6 (55)	24 (47)
Race, n (%)				
White	32 (100)	29 (97)	11 (100)	50 (98)
Asian	0 (0)	1 (3)	0 (0)	1 (2)
Age, median (range)	57 (20-72)	44 (23-72)	63 (25-72)	46 (20-72)
Age group at vaccination, n (%)				
18–55 yrs	14 (44)	22 (73)	3 (27)	33 (65)
56-85 yrs	18 (56)	8 (27)	8 (73)	18 (35)
Baseline SARS-CoV-2 status, n (%)				
Positive	0 (0)	0 (0)	0 (0)	0 (0)
Negative	32 (100)*	30 (100)[#]	11 (100)°	51 (100)[*, #,°]
Unknown	0 (0)	0 (0)	0 (0)	0 (0)
Interval, median (range)				
Days between D1/D2	21 (19-23)	‡	21 (19-23)	n/a
Days until serum draw after D2	22 (19-23)	n/a	21 (19-23)	n/a
Days between D2/D3	n/a	219 (180-342)	256 (180-342)	n/a
Days until 1m serum draw after D3	n/a	28 (26-30)	28 (28)	n/a

n/a, not available; D, Dose; Yrs, Years; m, Month; n, Number.
*, Negative SARS-CoV-2 PCR test at the time of enrollment
[#], No evidence of prior SARS-CoV-2 infection (based on COVID-19 symptoms/signs and SARS-CoV-2 PCR test)
°, No evidence of prior SARS-CoV-2 infection in the 12 weeks prior to enrollment
‡, A subset of participants received the primary series of BNT162b2 vaccine as part of a governmental vaccination program and the interval between doses was not recorded

Table S2. pVNT$_{50}$ values of 32 sera collected 21 days after the second dose of 30 µg BNT162b2.

Clinical trial	Participant ID	Participant age	pVNT$_{50}$			
			Wuhan	Omicron	Beta	Delta
BNT162-01	1	68	80	5	20	40
	2	57	160	5	10	40
	3	68	80	5	n/a	80
	4	62	80	5	10	40
	5	52	160	5	10	40
	6	57	320	5	n/a	n/a
	7	24	320	20	80	160
	8	62	20	5	5	5
	9	25	160	5	20	80
	10	47	160	10	40	80
	11	35	320	10	320	320
	12	51	160	5	20	80
	13	23	160	5	40	40
	14	26	160	10	20	80
	15	57	160	5	10	80
	16	55	160	5	160	80
	17	37	160	5	20	80
	18	40	640	10	40	160
	19	59	160	10	80	160
	20	29	160	5	20	80
	21	57	160	5	10	80
	22	70	40	5	5	10
	23	61	80	5	5	40
	24	65	160	10	20	160
	25	57	160	5	20	80
	26	63	320	10	40	160
	27	58	80	5	20	40
	28	36	80	5	10	40
	29	60	320	20	40	160
	30	72	320	10	40	160
	31	70	640	20	40	160
	32	20	320	20	80	160

n/a, not available due to lack of serum.

Table S3. pVNT$_{50}$ values of 30 sera collected 1 month after the third dose of 30 µg BNT162b2.

Clinical trial	Participant ID	Participant age	pVNT$_{50}$			
			Wuhan	Omicron	Beta	Delta
BNT162-17	33	49	5120	1280	1280	2560
	34	54	320	160	160	320
	35	35	640	320	80	640
	36	39	320	160	160	320
	37	36	320	160	320	320
	38	31	320	160	320	320
	39	23	320	160	160	640
	40	44	320	160	160	320
	41	51	40	5	20	20
	42	44	320	80	320	320
	43	37	160	80	160	320
	44	36	320	320	320	320
	45	38	160	40	160	160
	46	46	320	320	320	640
	47	30	640	160	320	320
	48	40	160	40	80	160
	49	37	1280	640	640	1280
	50	33	640	160	320	320
	51	44	2560	640	640	1280
BNT162-14	1	69	160	20	160	160
	2	58	320	320	1280	1280
	3	69	320	160	640	640
	4	63	320	160	320	320
	5	53	80	320	320	640
	6	58	640	160	320	640
	7	25	1280	1280	1280	1280
	8	63	40	40	40	40
	9	26	320	160	320	640
	30	72	640	160	640	640
	31	70	1280	640	1280	1280

Table S4. VNT_{50} values of 32 sera collected 21 days after the second dose of 30 µg BNT162b2.

Clinical trial	Participant ID	Participant age	VNT_{50}	
			Wuhan	Omicron
BNT162-01	1	68	80	5
	2	57	226	5
	3	68	226	5
	4	62	160	5
	5	52	453	5
	6	57	320	5
	7	24	1810	n/a
	8	62	40	5
	9	25	453	n/a
	10	47	905	n/a
	11	35	640	n/a
	12	51	320	n/a
	13	23	453	n/a
	14	26	320	n/a
	15	57	226	5
	16	55	320	5
	17	37	640	5
	18	40	320	7
	19	59	320	7
	20	29	640	5
	21	57	320	5
	22	70	226	5
	23	61	226	5
	24	65	640	10
	25	57	226	5
	26	63	453	7
	27	58	113	5
	28	36	160	5
	29	60	1280	20
	30	72	1280	10
	31	70	1810	14
	32	20	905	14

n/a, not available due to lack of serum.

Table S5. VNT50 values of 30 sera collected 1 month after the third dose of 30 µg BNT162b2.

Clinical trial	Participant ID	Participant age	VNT50	
			Wuhan	Omicron
BNT162-17	33	49	n/a	453
	34	54	n/a	40
	35	35	n/a	57
	36	39	n/a	80
	37	36	n/a	n/a
	38	31	n/a	160
	39	23	n/a	80
	40	44	n/a	80
	41	51	n/a	5
	42	44	n/a	40
	43	37	n/a	57
	44	36	n/a	160
	45	38	n/a	40
	46	46	n/a	226
	47	30	n/a	160
	48	40	n/a	28
	49	37	n/a	n/a
	50	33	n/a	113
	51	44	n/a	1280
BNT162-14	1	69	160	20
	2	58	1280	226
	3	69	320	160
	4	63	320	160
	5	53	453	160
	6	58	n/a	80
	7	25	n/a	905
	8	63	n/a	40
	9	26	n/a	226
	30	72	1280	160
	31	70	5120	320

n/a, not available due to lack of serum.

References and Notes

1. World Health Organization, Tracking SARS-CoV-2 variants (available at www.who.int/en/activities/tracking-SARS-CoV-2-variants/).

2. World Health Organization, SARS-CoV-2 Delta variant now dominant in much of European region; efforts must be reinforced to prevent transmission, warns WHO Regional Office for Europe and ECDC (available at www.euro.who.int/en/media-centre/sections/press-releases/2021/sars-cov-2-delta-variant-now-dominant-in-much-of-european-region-efforts-must-be-reinforced-to-prevent-transmission,-warns-who-regional-office-for-europe-and-ecdc).

3. WHO Technical Advisory Group on SARS-CoV-2 Virus Evolution (TAG-VE), Classification of Omicron (B.1.1.529): SARS-CoV-2 Variant of Concern (2021).

4. WHO Headquarters (HQ), WHO Health Emergencies Programme, Enhancing Readiness for Omicron (B.1.1.529): Technical Brief and Priority Actions for Member States (2021).

5. L. Premkumar, B. Segovia-Chumbez, R. Jadi, D. R. Martinez, R. Raut, A. Markmann, C. Cornaby, L. Bartelt, S. Weiss, Y. Park, C. E. Edwards, E. Weimer, E. M. Scherer, N. Rouphael, S. Edupuganti, D. Weiskopf, L. V. Tse, Y. J. Hou, D. Margolis, A. Sette, M. H. Collins, J. Schmitz, R. S. Baric, A. M. de Silva, The receptor binding domain of the viral spike protein is an immunodominant and highly specific target of antibodies in SARS-CoV-2 patients. *Sci. Immunol.* **5**, eabc8413 (2020). doi:10.1126/sciimmunol.abc8413 Medline

6. W. T. Harvey, A. M. Carabelli, B. Jackson, R. K. Gupta, E. C. Thomson, E. M. Harrison, C. Ludden, R. Reeve, A. Rambaut, S. J. Peacock, D. L. Robertson; COVID-19 Genomics UK (COG-UK) Consortium, SARS-CoV-2 variants, spike mutations and immune escape. *Nat. Rev. Microbiol.* **19**, 409–424 (2021). doi:10.1038/s41579-021-00573-0 Medline

7. P. Wang, M. S. Nair, L. Liu, S. Iketani, Y. Luo, Y. Guo, M. Wang, J. Yu, B. Zhang, P. D. Kwong, B. S. Graham, J. R. Mascola, J. Y. Chang, M. T. Yin, M. Sobieszczyk, C. A. Kyratsous, L. Shapiro, Z. Sheng, Y. Huang, D. D. Ho, Antibody resistance of SARS-CoV-2 variants B.1.351 and B.1.1.7. *Nature* **593**, 130–135 (2021). doi:10.1038/s41586-021-03398-2 Medline

8. D. Planas, D. Veyer, A. Baidaliuk, I. Staropoli, F. Guivel-Benhassine, M. M. Rajah, C. Planchais, F. Porrot, N. Robillard, J. Puech, M. Prot, F. Gallais, P. Gantner, A. Velay, J. Le Guen, N. Kassis-Chikhani, D. Edriss, L. Belec, A. Seve, L. Courtellemont, H. Péré, L. Hocqueloux, S. Fafi-Kremer, T. Prazuck, H. Mouquet, T. Bruel, E. Simon-Lorière, F. A. Rey, O. Schwartz, Reduced sensitivity of SARS-CoV-2 variant Delta to antibody neutralization. *Nature* **596**, 276–280 (2021). doi:10.1038/s41586-021-03777-9 Medline

9. Q. Wang, M. S. Nair, S. Anang, S. Zhang, H. Nguyen, Y. Huang, L. Liu, D. D. Ho, J. G. Sodroski, Functional differences among the spike glycoproteins of multiple emerging severe acute respiratory syndrome coronavirus 2 variants of concern. *iScience* **24**, 103393 (2021). doi:10.1016/j.isci.2021.103393 Medline

10. A. J. Greaney, T. N. Starr, P. Gilchuk, S. J. Zost, E. Binshtein, A. N. Loes, S. K. Hilton, J. Huddleston, R. Eguia, K. H. D. Crawford, A. S. Dingens, R. S. Nargi, R. E. Sutton, N. Suryadevara, P. W. Rothlauf, Z. Liu, S. P. J. Whelan, R. H. Carnahan, J. E. Crowe Jr., J.

D. Bloom, Complete Mapping of Mutations to the SARS-CoV-2 Spike Receptor-Binding Domain that Escape Antibody Recognition. *Cell Host Microbe* **29**, 44–57.e9 (2021). doi:10.1016/j.chom.2020.11.007 Medline

11. U. Sahin, A. Muik, I. Vogler, E. Derhovanessian, L. M. Kranz, M. Vormehr, J. Quandt, N. Bidmon, A. Ulges, A. Baum, K. E. Pascal, D. Maurus, S. Brachtendorf, V. Lörks, J. Sikorski, P. Koch, R. Hilker, D. Becker, A.-K. Eller, J. Grützner, M. Tonigold, C. Boesler, C. Rosenbaum, L. Heesen, M.-C. Kühnle, A. Poran, J. Z. Dong, U. Luxemburger, A. Kemmer-Brück, D. Langer, M. Bexon, S. Bolte, T. Palanche, A. Schultz, S. Baumann, A. J. Mahiny, G. Boros, J. Reinholz, G. T. Szabó, K. Karikó, P.-Y. Shi, C. Fontes-Garfias, J. L. Perez, M. Cutler, D. Cooper, C. A. Kyratsous, P. R. Dormitzer, K. U. Jansen, Ö. Türeci, BNT162b2 vaccine induces neutralizing antibodies and poly-specific T cells in humans. *Nature* **595**, 572–577 (2021). doi:10.1038/s41586-021-03653-6 Medline

12. F. P. Polack, S. J. Thomas, N. Kitchin, J. Absalon, A. Gurtman, S. Lockhart, J. L. Perez, G. Pérez Marc, E. D. Moreira, C. Zerbini, R. Bailey, K. A. Swanson, S. Roychoudhury, K. Koury, P. Li, W. V. Kalina, D. Cooper, R. W. Frenck Jr., L. L. Hammitt, Ö. Türeci, H. Nell, A. Schaefer, S. Ünal, D. B. Tresnan, S. Mather, P. R. Dormitzer, U. Şahin, K. U. Jansen, W. C. Gruber; C4591001 Clinical Trial Group, Safety and Efficacy of the BNT162b2 mRNA Covid-19 Vaccine. *N. Engl. J. Med.* **383**, 2603–2615 (2020). doi:10.1056/NEJMoa2034577 Medline

13. A. Muik, A.-K. Wallisch, B. Sänger, K. A. Swanson, J. Mühl, W. Chen, H. Cai, D. Maurus, R. Sarkar, Ö. Türeci, P. R. Dormitzer, U. Şahin, Neutralization of SARS-CoV-2 lineage B.1.1.7 pseudovirus by BNT162b2 vaccine-elicited human sera. *Science* **371**, 1152–1153 (2021). doi:10.1126/science.abg6105 Medline

14. J. Liu, Y. Liu, H. Xia, J. Zou, S. C. Weaver, K. A. Swanson, H. Cai, M. Cutler, D. Cooper, A. Muik, K. U. Jansen, U. Sahin, X. Xie, P. R. Dormitzer, P.-Y. Shi, BNT162b2-elicited neutralization of B.1.617 and other SARS-CoV-2 variants. *Nature* **596**, 273–275 (2021). doi:10.1038/s41586-021-03693-y Medline

15. Y. Liu, J. Liu, H. Xia, X. Zhang, C. R. Fontes-Garfias, K. A. Swanson, H. Cai, R. Sarkar, W. Chen, M. Cutler, D. Cooper, S. C. Weaver, A. Muik, U. Sahin, K. U. Jansen, X. Xie, P. R. Dormitzer, P.-Y. Shi, Neutralizing Activity of BNT162b2-Elicited Serum. *N. Engl. J. Med.* **384**, 1466–1468 (2021). doi:10.1056/NEJMc2102017 Medline

16. D. S. Khoury, D. Cromer, A. Reynaldi, T. E. Schlub, A. K. Wheatley, J. A. Juno, K. Subbarao, S. J. Kent, J. A. Triccas, M. P. Davenport, Neutralizing antibody levels are highly predictive of immune protection from symptomatic SARS-CoV-2 infection. *Nat. Med.* **27**, 1205–1211 (2021). doi:10.1038/s41591-021-01377-8 Medline

17. J. B. Case, P. W. Rothlauf, R. E. Chen, Z. Liu, H. Zhao, A. S. Kim, L.-M. Bloyet, Q. Zeng, S. Tahan, L. Droit, M. X. G. Ilagan, M. A. Tartell, G. Amarasinghe, J. P. Henderson, S. Miersch, M. Ustav, S. Sidhu, H. W. Virgin, D. Wang, S. Ding, D. Corti, E. S. Theel, D. H. Fremont, M. S. Diamond, S. P. J. Whelan, Neutralizing Antibody and Soluble ACE2 Inhibition of a Replication-Competent VSV-SARS-CoV-2 and a Clinical Isolate of SARS-CoV-2. *Cell Host Microbe* **28**, 475–485.e5 (2020). doi:10.1016/j.chom.2020.06.021 Medline

18. A. B. Vogel, I. Kanevsky, Y. Che, K. A. Swanson, A. Muik, M. Vormehr, L. M. Kranz, K. C. Walzer, S. Hein, A. Güler, J. Loschko, M. S. Maddur, A. Ota-Setlik, K. Tompkins, J. Cole, B. G. Lui, T. Ziegenhals, A. Plaschke, D. Eisel, S. C. Dany, S. Fesser, S. Erbar, F. Bates, D. Schneider, B. Jesionek, B. Sänger, A.-K. Wallisch, Y. Feuchter, H. Junginger, S. A. Krumm, A. P. Heinen, P. Adams-Quack, J. Schlereth, S. Schille, C. Kröner, R. de la Caridad Güimil Garcia, T. Hiller, L. Fischer, R. S. Sellers, S. Choudhary, O. Gonzalez, F. Vascotto, M. R. Gutman, J. A. Fontenot, S. Hall-Ursone, K. Brasky, M. C. Griffor, S. Han, A. A. H. Su, J. A. Lees, N. L. Nedoma, E. H. Mashalidis, P. V. Sahasrabudhe, C. Y. Tan, D. Pavliakova, G. Singh, C. Fontes-Garfias, M. Pride, I. L. Scully, T. Ciolino, J. Obregon, M. Gazi, R. Carrion Jr., K. J. Alfson, W. V. Kalina, D. Kaushal, P.-Y. Shi, T. Klamp, C. Rosenbaum, A. N. Kuhn, Ö. Türeci, P. R. Dormitzer, K. U. Jansen, U. Sahin, BNT162b vaccines protect rhesus macaques from SARS-CoV-2. *Nature* **592**, 283–289 (2021). doi:10.1038/s41586-021-03275-y Medline

19. L. J. Abu-Raddad, H. Chemaitelly, A. A. Butt; National Study Group for COVID-19 Vaccination, Effectiveness of the BNT162b2 Covid-19 Vaccine against the B.1.1.7 and B.1.351 Variants. *N. Engl. J. Med.* **385**, 187–189 (2021). doi:10.1056/NEJMc2104974 Medline

20. O. Mor, N. S. Zuckerman, I. Hazan, R. Fluss, N. Ash, N. Ginish, E. Mendelson, S. Alroy-Preis, L. Freedman, A. Huppert, BNT162b2 vaccine effectiveness was marginally affected by the SARS-CoV-2 beta variant in fully vaccinated individuals. *J. Clin. Epidemiol.* **142**, 38–44 (2022). doi:10.1016/j.jclinepi.2021.10.011 Medline

21. A. Kuzmina, S. Wattad, Y. Khalaila, A. Ottolenghi, B. Rosental, S. Engel, E. Rosenberg, R. Taube, SARS CoV-2 Delta variant exhibits enhanced infectivity and a minor decrease in neutralization sensitivity to convalescent or post-vaccination sera. *iScience* **24**, 103467 (2021). doi:10.1016/j.isci.2021.103467 Medline

22. S. Cele, L. Jackson, D. S. Khoury, K. Khan, T. Moyo-Gwete, H. Tegally, J. E. San, D. Cromer, C. Scheepers, D. Amoako, F. Karim, M. Bernstein, G. Lustig, D. Archary, M. Smith, Y. Ganga, Z. Jule, K. Reedoy, S.-H. Hwa, J. Giandhari, J. M. Blackburn, B. I. Gosnell, S. S. Abdool Karim, W. Hanekom, NGS-SA, COMMIT-KZN Team, A. von Gottberg, J. Bhiman, R. J. Lessells, M.-Y. S. Moosa, M. P. Davenport, T. de Oliveira, P. L. Moore, A. Sigal, SARS-CoV-2 Omicron has extensive but incomplete escape of Pfizer BNT162b2 elicited neutralization and requires ACE2 for infection. medRxiv 2021.12.08.21267417 (2021); https://doi.org/10.1101/2021.12.08.21267417.

23. A. Wilhelm, M. Widera, K. Grikscheit, T. Toptan, B. Schenk, C. Pallas, M. Metzler, N. Kohmer, S. Hoehl, F. A. Helfritz, T. Wolf, U. Goetsch, S. Ciesek, Reduced Neutralization of SARS-CoV-2 Omicron Variant by Vaccine Sera and Monoclonal Antibodies. medRxiv 2021.12.07.21267432 (2021); https://doi.org/10.1101/2021.12.07.21267432.

24. A. R. Falsey, R. W. Frenck Jr., E. E. Walsh, N. Kitchin, J. Absalon, A. Gurtman, S. Lockhart, R. Bailey, K. A. Swanson, X. Xu, K. Koury, W. Kalina, D. Cooper, J. Zou, X. Xie, H. Xia, Ö. Türeci, E. Lagkadinou, K. R. Tompkins, P.-Y. Shi, K. U. Jansen, U. Şahin, P. R. Dormitzer, W. C. Gruber, SARS-CoV-2 Neutralization with BNT162b2 Vaccine Dose 3. *N. Engl. J. Med.* **385**, 1627–1629 (2021). doi:10.1056/NEJMc2113468 Medline

25. B. Grunau, D. M. Goldfarb, M. Asamoah-Boaheng, L. Golding, T. L. Kirkham, P. A. Demers, P. M. Lavoie, Immunogenicity of Extended mRNA SARS-CoV-2 Vaccine Dosing Intervals. *JAMA* (2021). doi:10.1001/jama.2021.21921 Medline

26. A. Bertoletti, N. Le Bert, M. Qui, A. T. Tan, SARS-CoV-2-specific T cells in infection and vaccination. *Cell. Mol. Immunol.* **18**, 2307–2312 (2021). doi:10.1038/s41423-021-00743-3 Medline

27. UK Health Security Agency, SARS-CoV-2 variants of concern and variants under investigation in England - Technical briefing: Update on hospitalisation and vaccine effectiveness for Omicron VOC-21NOV-01 (B.1.1.529) (available at https://assets.publishing.service.gov.uk/government/uploads/system/uploads/attachment_data/file/1044481/Technical-Briefing-31-Dec-2021-Omicron_severity_update.pdf).

28. N. Ferguson *et al.*, Report 49: Growth, population distribution and immune escape of Omicron in England (2021) (available at www.imperial.ac.uk/media/imperial-college/medicine/mrc-gida/2021-12-16-COVID19-Report-49.pdf).

29. S. Khare, C. Gurry, L. Freitas, M. B. Schultz, G. Bach, A. Diallo, N. Akite, J. Ho, R. T. Lee, W. Yeo, GISAID Core Curation Team, S. Maurer-Stroh, GISAID's Role in Pandemic Response. *China CDC Weekly* **3**, 1049–1051 (2021). doi:10.46234/ccdcw2021.255 Medline

30. K. Katoh, D. M. Standley, MAFFT multiple sequence alignment software version 7: Improvements in performance and usability. *Mol. Biol. Evol.* **30**, 772–780 (2013). doi:10.1093/molbev/mst010 Medline

31. M. Berger Rentsch, G. Zimmer, A vesicular stomatitis virus replicon-based bioassay for the rapid and sensitive determination of multi-species type I interferon. *PLOS ONE* **6**, e25858 (2011). doi:10.1371/journal.pone.0025858 Medline

32. W. Fleri, N. Salimi, R. Vita, B. Peters, A. Sette, Immune Epitope Database and Analysis Resource (2016). *Encyclopedia of Immunobiology*. **2** (2016).
