# Supplementary material for: Neutralization of SARS-CoV-2 Omicron by BNT162b2 mRNA vaccine–elicited human sera
Source: Science. 2022 Jan 18;375(6581):678–80. doi: 10.1126/science.abn7591 (PMC9836206; doi:10.1126/science.abn7591)
Supplement: Supplementary file 3 — MDAR Reproducibility Checklist [file science.abn7591_mdar_reproducibility_checklist.pdf]

Materials Design Analysis Reporting (MDAR)
Checklist for Authors

The MDAR framework establishes a minimum set of requirements in transparent reporting applicable to studies in the life sciences (see Statement of Task: doi:10.31222/osf.io/9sm4x.). The MDAR checklist is a tool for authors, editors, and others seeking to adopt the MDAR framework for transparent reporting in manuscripts and other outputs. Please refer to the MDAR Elaboration Document for additional context for the MDAR framework.

For all that apply, please note where in the manuscript the required information is provided.

Materials:

Newly created materials	indicate where provided: page no/section/legend)	n/a
The manuscript includes a dedicated "materials availability statement" providing transparent disclosure about availability of newly created materials including details on how materials can be accessed and describing any restrictions on access.	Page 15 (Data and materials availability)<br><br>Materials are available from the authors under a material transfer agreement with BioNTech.	

Antibodies	indicate where provided: page no/section/legend)	n/a
For commercial reagents, provide supplier name, catalogue number and RRID, if available.	Supplementary Materials, page 4-5<br>(Materials and Methods – VSV-SARS-CoV-2 S variant pseudovirus generation)<br><br>anti-VSV-G antibody (clone 8G5F11, Kerafast Inc.)	

DNA and RNA sequences	indicate where provided: page no/section/legend)	n/a
**Short novel DNA or RNA including primers, probes:** Sequences should be included or deposited in a public repository.		X

Cell materials	indicate where provided: page no/section/legend	n/a
**Cell lines:** Provide species information, strain. Provide accession number in repository **OR** supplier name, catalog number, clone number, **OR** RRID.	Supplementary Materials, page 5 and 7<br>(Materials and Methods – VSV-SARS-CoV-2 S variant pseudovirus generation / Live SARS-CoV-2 neutralization assay)<br><br>HEK293T/17 (ATCC® CRL-11268™)<br>VERO 76 (ATCC® CRL-1587™)<br>Vero E6 (ATCC® CRL-1586™)	
**Primary cultures:** Provide species, strain, sex of origin, genetic modification status.		X

Experimental animals	indicate where provided: page no/section/legend)	n/a
**Laboratory animals or Model organisms:** Provide species, strain, sex, age, genetic modification status. Provide accession number in repository **OR** supplier name, catalog number, clone number, **OR** RRID.		X
**Animal observed in or captured from the field:** Provide species, sex, and age where possible.		X

Plants and microbes	indicate where provided: page no/section/legend)	n/a
**Plants:** provide species and strain, ecotype and cultivar where relevant, unique accession number if available, and source (including location for collected wild specimens).		X
**Microbes:** provide species and strain, unique accession number if available, and source.		X

Human research participants	indicate where provided: page no/section/legend) or state if these demographics were not collected	n/a
If collected and within the bounds of privacy constraints report on age, sex and gender or ethnicity for all study participants.	Supplementary Materials, pages 2-3<br>(Materials and Methods – Clinical Trials)	

	This research used samples from 18-85 year old participants of the German Phase 1/2 trial BNT162-01 (NCT04380701) vaccinated with 2-dose primary series of 30 µg BNT162b2 with a 21 ± 2 days dosing interval. In addition, samples were used from participants in subgroups of two ongoing clinical trials, BNT162-14 (NCT04949490) aged 18-85 and BNT162-17 (NCT05004181) aged 18-55 years old.<br>Participants of BNT162-01 who received the 2-dose primary series with BNT162b2 were recruited to the BNT162-14 clinical trial, a Phase 2, open-label, rollover trial located at multiple sites in Germany.<br>Participants included in this study from these clinical trials were from subcohorts contributing to the exploratory endpoint to evaluate cross-neutralization of BNT162b2-induced antibodies to emerging SARS-CoV-2 variants following 2-dose primary series vaccination with or without booster vaccination (dose 3) in healthy adults.<br>The trials were carried out in accordance with the Declaration of Helsinki and Good Clinical Practice Guidelines and with approval by independent ethics committees and the competent regulatory authorities. All participants provided written informed consent. The primary objectives of these trials will be reported at a later date.<br><br>Table S1. Cohort characteristics (page 14)	

Design:

**Study protocol**	**indicate where provided: page no/section/legend)**	**n/a**
If study protocol has been pre-registered, provide DOI. For clinical trials, provide the trial registration number **OR** cite DOI.		X

**Laboratory protocol**	**indicate where provided: page no/section/legend)**	**n/a**
Provide DOI **OR** other citation details if detailed step-by-step protocols are available.		X

**Experimental study design (statistics details)**		
**For in vivo studies:** State whether and how the following have been done	**indicate where provided: page no/section/legend. If it could have been done, but was not, write not done**	**n/a**
Sample size determination		X
Randomisation		X
Blinding		X
Inclusion/exclusion criteria		X

**Sample definition and in-laboratory replication**	**indicate where provided: page no/section/legend**	**n/a**
State number of times the experiment was replicated in laboratory.	Supplementary Materials, page 6 (Materials and Methods – Pseudovirus neutralization assay)<br><br>The full set of sera was tested for neutralization of SARS-CoV-2 pseudoviruses in three independent assays.	
Define whether data describe technical or biological replicates.	Supplementary Materials, page 3, 6-7 (Materials and Methods – Serum specimens / Pseudovirus neutralization assay / Live SARS-CoV-2 neutralization assay)<br><br>Three different panels of sera were investigated. The first serum panel was obtained from 32 participants in the BNT162-01 trial drawn at a median 22 days (range 19-23 days) after receiving the second dose BNT162b2 of the 2-dose primary series (2 x 30µg). The second serum panel was obtained from 30 participants in the BNT162-14 (n=11) and BNT162-17 (n=19) trial drawn at a median 28 days (range 26-30 days) after receiving the third dose BNT162b2. The third serum panel was obtained from 11 participants immunized with a third dose of BNT162b2 in the BNT162-14 trial, who rolled over from the parental trial BNT162-01.<br><br>Serum dilutions were mixed 1:1 with pseudovirus (n=2 technical replicates per serum per pseudovirus) for 30 minutes at room temperature prior to addition to Vero 76 cell monolayers and incubation at 37 °C with 7.5% CO_2 for 24 hours.	

**Ethics**	**indicate where provided: page no/section/legend**	**n/a**

**Studies involving human participants:** State details of authority granting ethics approval (IRB or equivalent committee(s), provide reference number for approval.		X
**Studies involving experimental animals:** State details of authority granting ethics approval (IRB or equivalent committee(s), provide reference number for approval.		X
**Studies involving specimen and field samples:** State if relevant permits obtained, provide details of authority approving study; if none were required, explain why.	Supplementary Materials, pages 2-3 (Materials and Methods – Clinical Trials)<br><br>This research used samples from 18-85 year old participants of the German Phase 1/2 trial BNT162-01 (NCT04380701) vaccinated with 2-dose primary series of 30 µg BNT162b2 with a 21 ± 2 days dosing interval. In addition, samples were used from participants in subgroups of two ongoing clinical trials, BNT162-14 (NCT04949490) aged 18-85 and BNT162-17 (NCT05004181) aged 18-55 years old.<br>Participants of BNT162-01 who received the 2-dose primary series with BNT162b2 were recruited to the BNT162-14 clinical trial, a Phase 2, open-label, rollover trial located at multiple sites in Germany.<br>Participants included in this study from these clinical trials were from subcohorts contributing to the exploratory endpoint to evaluate cross-neutralization of BNT162b2-induced antibodies to emerging SARS-CoV-2 variants following 2-dose primary series vaccination with or without booster vaccination (dose 3) in healthy adults.<br>The trials were carried out in accordance with the Declaration of Helsinki and Good Clinical Practice Guidelines and with approval by independent ethics committees and the competent regulatory authorities. All participants provided written informed consent. The primary objectives of these trials will be reported at a later date.	

Dual Use Research of Concern (DURC)	indicate where provided: page no/section/legend	n/a
If study is subject to dual use research of concern regulations, state the authority granting approval and reference number for the regulatory approval.		X

Analysis:

Attrition	indicate where provided: page no/section/legend	n/a
Describe whether exclusion criteria were preestablished. Report if sample or data points were omitted from analysis. If yes report if this was due to attrition or intentional exclusion and provide justification.		X

Statistics	indicate where provided: page no/section/legend	n/a
Describe statistical tests used and justify choice of tests.	Supplementary Materials, page 8<br>(Materials and Methods – Statistical analysis):<br><br>The statistical method of aggregation used for the analysis of antibody titers is the geometric mean and the corresponding 95% confidence interval. Using the geometric mean accounts for non-normal distribution of antibody titers that span several orders of magnitude. Spearman correlation was used to evaluate the monotonic relationship between non-normally distributed datasets.	

Data availability	indicate where provided: page no/section/legend	n/a
For newly created and reused datasets, the manuscript includes a data availability statement that provides details for access or notes restrictions on access.	Section – Data and material availability, page 15	
If newly created datasets are publicly available, provide accession number in repository **OR** DOI **OR** URL and licensing details where available.		X
If reused data is publicly available provide accession number in repository **OR** DOI **OR** URL, **OR** citation.		X

Code availability	indicate where provided: page no/section/legend	n/a
For all newly generated custom computer code/software/mathematical algorithm or re-used code essential for replicating the main findings of the study, the manuscript includes a data availability statement that provides details for access or notes restrictions.		X
If newly generated code is publicly available, provide accession number in repository, **OR** DOI **OR** URL and licensing details where available. State any restrictions on code availability or accessibility.		X
If reused code is publicly available provide accession number in repository **OR** DOI **OR** URL, **OR** citation.		X

Reporting

MDAR framework recommends adoption of discipline-specific guidelines, established and endorsed through community initiatives. Journals have their own policy about requiring specific guidelines and recommendations to complement MDAR.

Adherence to community standards	indicate where provided: page no/section/legend	n/a
State if relevant guidelines (e.g., ICMJE, MIBBI, ARRIVE) have been followed, and whether a checklist (e.g., CONSORT, PRISMA, ARRIVE) is provided with the manuscript.		X
